# ﻿A survey of *Orchestina* Simon, 1882 (Araneae, Oonopidae) from Xishuangbanna, China, with descriptions of eight new species

**DOI:** 10.3897/zookeys.1249.163886

**Published:** 2025-08-25

**Authors:** Chenxue Song, Dongju Bian, Yanfeng Tong, Shuqiang Li

**Affiliations:** 1 College of Life Science, Shenyang Normal University, Shenyang 110034, Liaoning, China Shenyang Normal University Shenyang China; 2 CAS Key Laboratory of Forest Ecology and Silviculture, Institute of Applied Ecology, Chinese Academy of Sciences, Shenyang 110016, China Chinese Academy of Sciences Shenyang China; 3 College of Life Sciences, Anhui Normal University, Wuhu, Anhui 241000, China Anhui Normal University Wuhu China

**Keywords:** Asia, goblin spiders, identification key, Orchestininae, taxonomy

## Abstract

Eight new species and one known species of the oonopid spider genus *Orchestina* Simon, 1882 are described or recorded based on material collected from forest canopy of Xishuangbanna, Yunnan Province, southwestern China: *O. alata***sp. nov.** (♂♀), *O. aureola* Tong & Li, 2011 (♂♀), *O. caixiaae* Tong & Li, **sp. nov.** (♂♀), *O. longituba* Tong & Li, **sp. nov.** (♂♀), *O. qingyuani* Tong & Li, **sp. nov.** (♂♀), *O. subconcava* Tong & Li, **sp. nov.** (♂♀), *O. sublongituba* Tong & Li, **sp. nov.** (♂♀), *O. tentoria* Tong & Li, **sp. nov.** (♂♀) and *O. xuexing* Tong & Li, **sp. nov.** (♂♀). An identification key to these nine species is provided.

## ﻿Introduction

Goblin spiders (Oonopidae Simon, 1890) are a family of tiny spiders (body length 1.0–3.0 mm), currently comprising 1968 extant described species in 115 genera globally (WSC 2025). *Orchestina* Simon, 1882 is a species-rich genus that currently contains 173 extant species (WSC 2025). It has an almost global distribution and occurs in the Northern Hemisphere in the region south of 45°N (Marusik et al. 2018). This genus is considered a dominant group in canopies (Tong and Li 2011; Henrard and Jocqué 2012). Occasionally, some of these spiders are also found under bark, in litter, grasses, and suspended litter, and even inside buildings (Izquierdo and Ramírez 2017). In China, 24 species of *Orchestina* have been reported to date (Song et al. 2024; Wang et al. 2024; Ma et al. 2025; Zhou et al. 2025).

Xishuangbanna, situated in southern Yunnan Province, harbors the most well-preserved tropical rainforest in China and is part of the Indo-Burma biodiversity hotspot (Myers 1988). A recent survey identified eight *Orchestina* species in this region. Notably, all previously known *Orchestina* species from Xishuangbanna exhibit a distinctive net-shaped pattern on their carapaces (Song et al. 2024). While studying spiders collected from forest canopy of Xishuangbanna, we found many *Orchestina* specimens lacking any carapace patterns. The present paper aims to provide the descriptions of eight new species and one known species from this region.

## ﻿Materials and methods

The specimens were examined using a Leica M205 C stereomicroscope. Details of body parts and measurements were studied under an Olympus BX51 compound microscope. Endogynes were cleared in lactic acid. Photomicroscope images were taken with a Canon EOS 750D zoom digital camera (18 megapixels) mounted on the Olympus BX51. Raw photos were first stacked with Helicon Focus v. 8.2.0 to obtain the composite images, which were then processed in Adobe Photoshop 21.1.2. All measurements in the text are given in millimeters. Terminology and taxonomic descriptions follow Henrard and Jocqué (2012) and Tong and Li (2011). All material studied is deposited in the Shenyang Normal University (**SYNU**) in Shenyang, China.

The following abbreviations are used in the text and figures: ARe = anterior receptaculum; AUS = anterior uterine sclerite; Exc = excavations; PME = posterior median eyes; PP = posterior plate; Pr = protrusion; XNNR = Xishuangbanna National Natural Reserve; XTBG = Xishuangbanna Tropical Botanical Garden.

## ﻿Taxonomy

**Family Oonopidae Simon, 1890**

**Genus *Orchestina* Simon, 1882**

### ﻿Key to species lacking carapace patterns of *Orchestina* from Xishuangbanna

For the key to species with carapace patterns, see Song et al. 2024.

**Table:** 

1	Male	**2**
–	Female	**10**
2	Palpal tibia not enlarged (Figs 3A, 9A, 18A)	**3**
–	Palpal tibia strongly enlarged (Figs 6A, 12A, 15A, 21A, 24A, 27A)	**5**
3	Sperm duct abruptly bent in the middle section, with 2 loops in prolateral view (Fig. 9A)	***O. caixiaae* sp. nov.**
–	Sperm duct not bent in the middle section, with at least 3 loops in prolateral view (Figs 3A, 18A)	**4**
4	Labium diamond-shaped; endites with smoothly curved outer margin; embolus with sub-apical wing-shaped protrusions (Fig. 3C, D)	***O. alata* sp. nov.**
–	Labium rounded; endites with deep excavation on outer margin; embolus with sub-apical crest and wrinkle (Fig. 18C, D)	***O. subconcava* sp. nov.**
5	Sperm duct with 5 loops (Fig. 24A)	***O. tentoria* sp. nov.**
–	Sperm duct with no more than 3 loops (e.g., Fig. 6A)	**6**
6	Sperm duct with 3 loops (Fig. 6A)	***O. aureola***
–	Sperm duct with 2 loops (e.g., Fig. 12A)	**7**
7	Embolus boot-shaped in prolateral view (Fig. 27A)	***O. xuexing* sp. nov.**
–	Embolus long tube-shaped in prolateral view (Figs 12A, 15D, 21A)	**8**
8	Sperm duct far away the edge of ventral side of bulb (Figs 12A, 21A); labium diamond-shaped or tongue-shaped (Figs 12C, 21C)	**9**
–	Sperm duct very close to the edge of ventral side of bulb (Fig. 15A); labium rectangular, with anterior margin anteriorly projecting at middle (Fig. 15C)	***O. qingyuani* sp. nov.**
9	Palpal tibia narrower than bulbus (Fig. 12A, B); labium diamond-shaped (Fig. 12C)	***O. longituba* sp. nov.**
–	Palpal tibia wider than bulbus (Fig. 21A, B); labium tongue-shaped	***O. sublongituba* sp. nov.**
10	Epigaster with a tent-shaped marking (Fig. 23H)	***O. tentoria* sp. nov.**
–	Without above mentioned character	**11**
11	Epigaster with a half tent-shaped marking (Fig. 17H)	***O. subconcava* sp. nov.**
–	Without above mentioned character	**12**
12	Epigaster with a saddle-shaped marking (Fig. 20H)	***O. sublongituba* sp. nov.**
–	Without above mentioned character	**13**
13	Anterior receptaculum distinctly longer than medial clavate sclerite (Fig. 6F)	***O. aureola***
–	Anterior receptaculum slightly longer than medial clavate sclerite (e.g., Fig. 3G)	**14**
14	Anterior receptaculum rounded (Fig. 15F)	***O. qingyuani* sp. nov.**
–	Anterior receptaculum triangular (e.g., Fig. 3G)	**15**
15	Lateral protrusions straight (Figs 3G, 9F)	**16**
–	Lateral protrusions oblique (Figs 12F, 27F)	**17**
16	Lateral protrusions longer than the width of medial clavate sclerite (Fig. 3G)	***O. alata* sp. nov.**
–	Lateral protrusions distinctly shorter than the width of medial clavate sclerite (Fig. 9F)	***O. caixiaae* sp. nov.**
17	Epigaster with distinct marking (Fig. 11H); lateral protrusions at angle of about 60° with medial clavate sclerite (Fig. 12F)	***O. longituba* sp. nov.**
–	Epigaster without distinct marking (Fig. 26H); lateral protrusions at angle of about 75° with medial clavate sclerite (Fig. 27F)	***O. xuexing* sp. nov.**

### ﻿Species descriptions

#### ﻿ Orchestina alata

Taxon classificationAnimalia

Tong & Li sp. nov.

2D3046E6-F65D-5C44-BA04-BF9846F2589C

https://zoobank.org/A0157007-8711-44D2-ACB9-0F56328651D8

Figs 1 , 2 , 3

##### Material examined.

***Holotype*** China • ♂ (SYNU-1604), fogging; Yunnan, Mengla Co., Menglun Town, XTBG, primary tropical seasonal rain forest; 21°55.035'N, 101°16.500'E, 558 ± 17 m; 22.VII.2007; Zheng G. leg. ***Paratypes*.** China • 1 ♂ (SYNU-1605), same data as holotype • 1 ♂ 1 ♀ (SYNU-1477–78), same data as holotype • 2 ♂ 1 ♀ (SYNU-1418–20), same data as holotype • 1 ♀ (SYNU-F–4756), fogging; Nanshahe Vill., seasonal rainforest; 21°36.201'N, 101°34.398'E, 826 ± 43 m; 14.VII.2012; Zhao Q. & Chen Z. leg.

**Figure 1.**
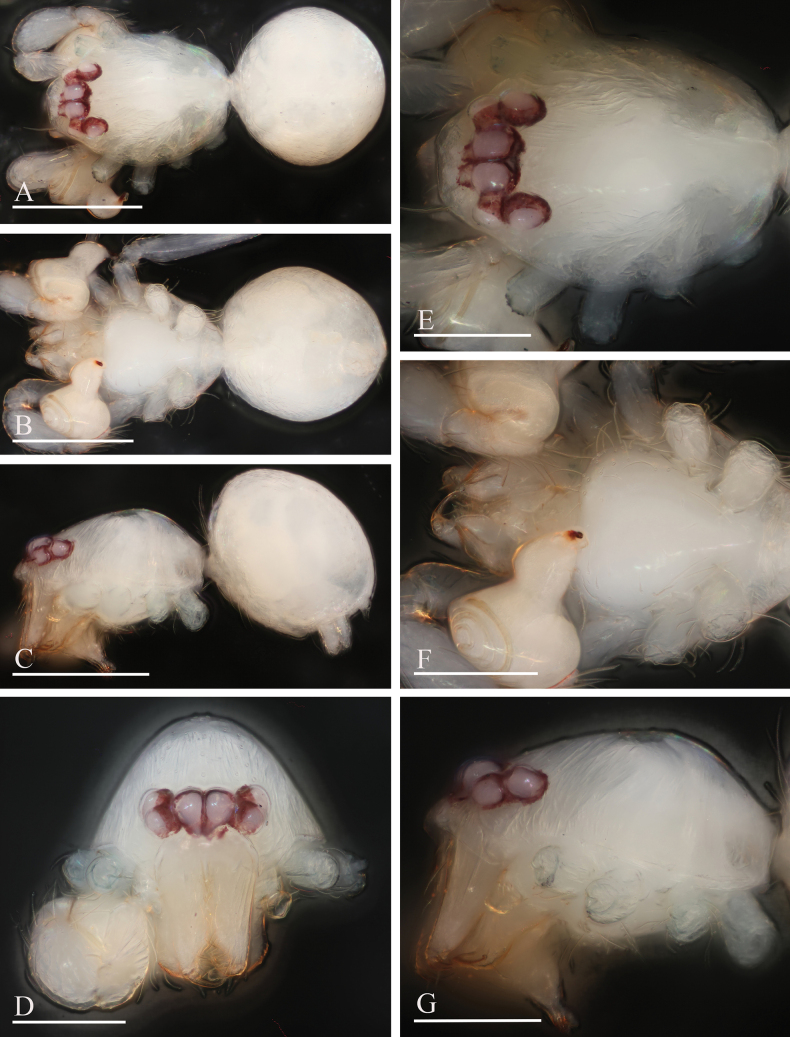
*Orchestina alata* sp. nov., holotype male. A–C. Habitus, dorsal, ventral and lateral views; D–G. Prosoma, anterior, dorsal, ventral and lateral views. Scale bars: 0.4 mm (A–C); 0.2 mm (D–G).

**Figure 2.**
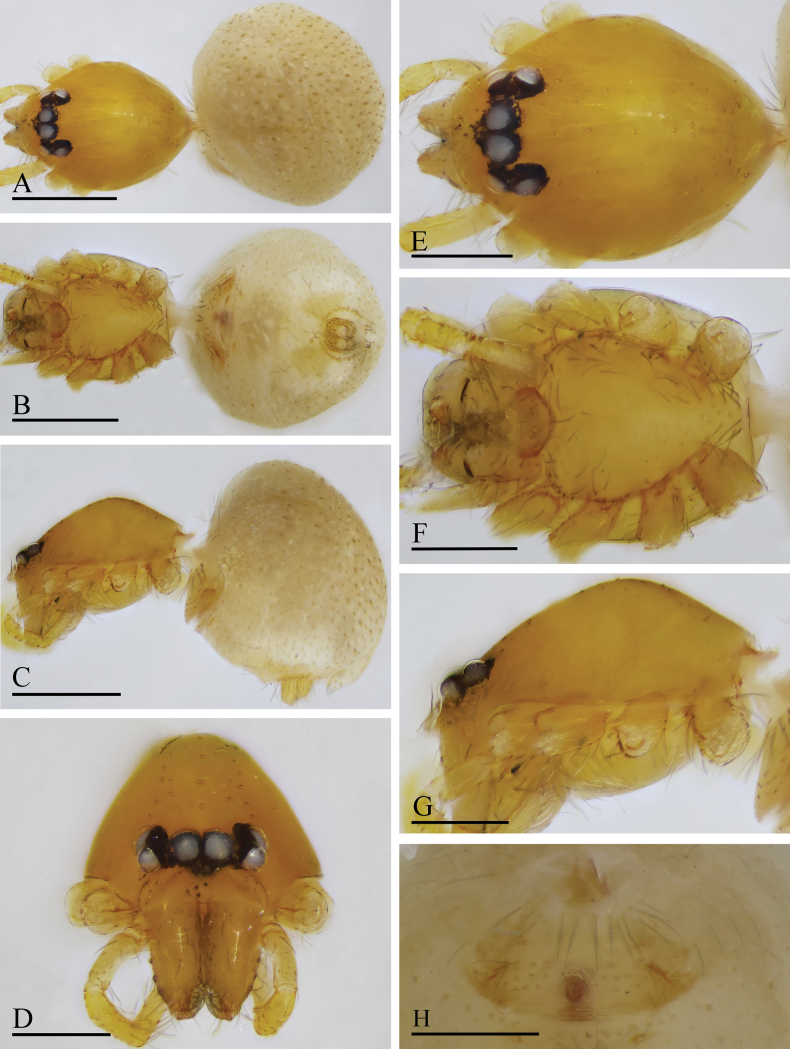
*Orchestina alata* sp. nov., paratype female. A–C. Habitus, dorsal, ventral and lateral views; D–G. Prosoma, anterior, dorsal, ventral and lateral views; H. Epigaster, ventral view. Scale bars: 0.4 mm (A–C); 0.2 mm (D–H).

##### Diagnosis.

**Figure 3.**
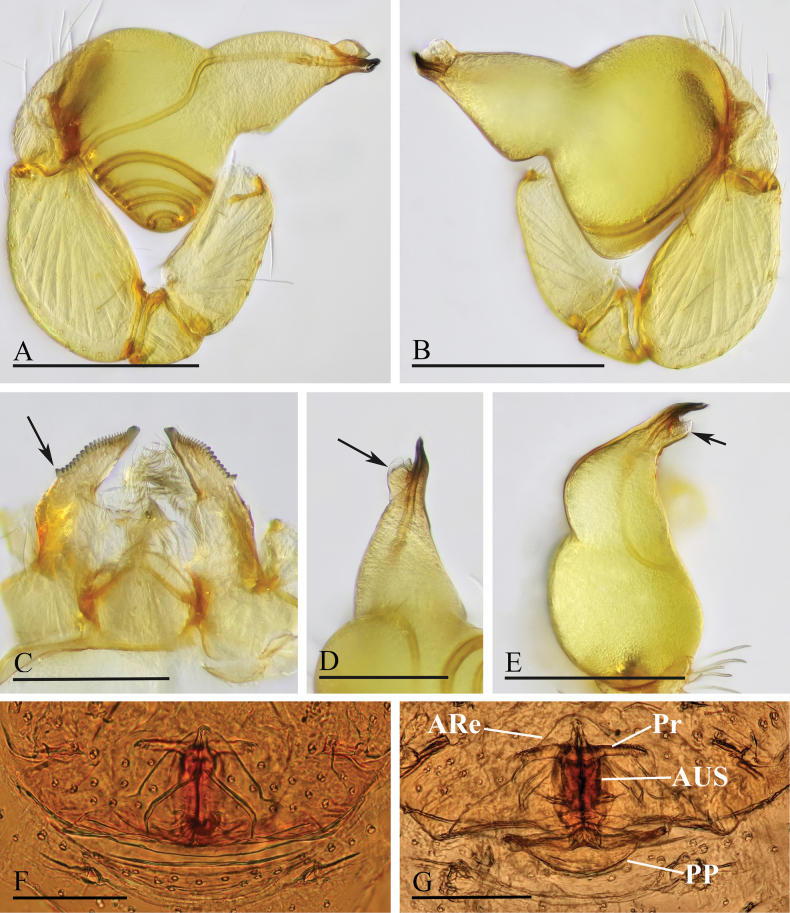
*Orchestina alata* sp. nov. A, B, E. Left palp, prolateral, retrolateral and dorsal views; C. Endites and labium, ventral view, arrow shows the large denticle; D. Distal part of bulb, prolateral view, arrow shows the wing-shaped protrusions; F, G. Endogyne, ventral and dorsal views. Abbreviations: ARe = anterior receptaculum; AUS = anterior uterine sclerite; PP = posterior plate; Pr = protrusion. Scale bars: 0.2 mm (A, B, E); 0.1 mm (C, D, F, G).

The new species is similar to *Orchestina sinensis* Xu, 1987 in the yellowish carapace and the pear-shaped bulbus, but can be distinguished by the sub-apical wing-shaped protrusions of bulbus (vs lacking, but with a crest; cf. Fig. 3A, D and Xu 1987: figs 5, 6), the basal large denticle of male endites (vs lacking; cf. Fig. 3C) and the short column-shaped marking of epigaster (vs with a large reddish oval spot; cf. Fig. 2H and Xu 1987: fig. 4).

##### Description.

**Male** (holotype). ***Carapace*** (Fig. 1A, D): 0.61 long, 0.41 wide; yellowish, oval in dorsal view, with some long setae; pars cephalica elevated in lateral view. ***Eyes*** (Fig. 1A, C): well developed, PME largest; posterior eye row recurved from dorsal view. ***Clypeus*** (Fig. 1C, D): margin unmodified, curved downwards in front view, sloping forward in lateral view. ***Sternum*** (Fig. 1B): as long as wide, surface smooth, setae sparse, needle-like, evenly scattered. ***Mouthparts*** (Figs 1B, C, 3C): chelicerae straight, anterior face unmodified; labium diamond-shaped, not fused to sternum, anterior margin not indented at middle; endites strongly sclerotized on outer margin, with serrula in single row, outer margin smoothly curved. ***Abdomen*** ovoid, 0.53 long; dorsum soft portions pale white, without color pattern. ***Legs***: yellow, without color pattern; femur IV thickened, wider than femora I−III. ***Palp*** (Fig. 3A–E): tibia not enlarged, length/width = 1.77, cymbium elongated ovoid; bulb pear-shaped in lateral view, with ventral side strongly protruding proximally, ca 1.72× as wide as tibia; the sperm duct with four loops in prolateral view; embolus tapered, with sub-apical wing-shaped protrusions.

**Female** (SYNU-1478). Same as male except as noted. ***Body***: habitus as in Fig. 2A–C; body length 1.36. ***Carapace*** (Fig. 2E): 0.66 long, 0.49 wide. ***Abdomen***: 0.69 long. ***Epigaster*** (Fig. 2H): without special external features.

***Endogyne*** (Fig. 3F, G): with stout medial clavate sclerite (AUS), provided with pair of lateral protrusions (Pr); anterior receptaculum (ARe) triangular, semitransparent, slightly longer than AUS; posterior plate (PP) present, large.

##### Distribution.

Known only from the type locality.

##### Etymology.

The specific name is derived from the Latin alatus, meaning ‘winged’, and refers to the subapical wing-shaped protrusions of the bulbus.

#### ﻿ Orchestina aureola

Taxon classificationAnimalia

Tong & Li, 2011

87C31BA4-DC83-5A8F-93BF-7693699B74DC

Figs 4 , 5 , 6

Orchestina aureola Tong & Li, 2011: 37, figs 1A, B, 2A, 3A, 4A, 5A–C, 6A–F; Tong 2013: 45, figs 26A–C, 27A, 28A, 29A, 30A–C, 64A–F.

##### Material examined.

China • 3 ♂ (SYNU-1591–93), fogging; Yunnan, Mengla Co., Menglun Town, XNNR, 48 Km.; 21°58.704'N, 101°19.748'E, 1088 ± 12 m; 12.VIII.2011; Zheng G., Zhao Q. & Gao C. leg. • 3 ♂ (SYNU-F-3960–62), same data as above • 8 ♂ (SYNU-F-3974–81), same data as above • 5 ♂ (SYNU-F-3982–86), same data as above • 2 ♀ (SYNU-1673–74), same data as above • 2 ♂ (SYNU-F-3958–59), fogging; XNNR, 55 Km., about 1 km up the hill, seasonal rainforest; 21°57.935'N, 101°12.305'E, 781 ± 17 m; 13.VIII.2011; Zheng G., Zhao Q. & Gao C. leg. • 8 ♂ (SYNU-F-3966–73), same data as above • 8 ♂ (SYNU-F-3987–94), fogging; XNNR, 55 Km., the valley forest beside the artificial fishpond; 21°57.883'N, 101°12.147'E, 839 ± 18 m; 15.VIII.2011; Zheng G., Zhao Q. & Gao C. leg. • 3 ♂ (SYNU-F-3995–97), fogging; XNNR, 200 m east of the Lvshilin, limestone monsoon rainforest; 21°54.617'N, 101°16.843'E, 738 ± 17 m; 7.VIII.2011; Zheng G., Zhao Q. & Gao C. leg. • 8 ♀ (SYNU-F-4770–4777), same data as above; 21°24.389'N, 101°16.754'E, 705 ± 21 m; 5.VIII.2011 • 1 ♀ (SYNU-F-4778), fogging; Huigang Vill., Xilu habitat restoration area, monsoon forest; 21°37.045'N, 101°35.268'E, 764 ± 25 m; 12.VII.2012; Zhao Q. & Chen Z. leg. • 5 ♀ (SYNU-F-4803–07), same data as above.

**Figure 4.**
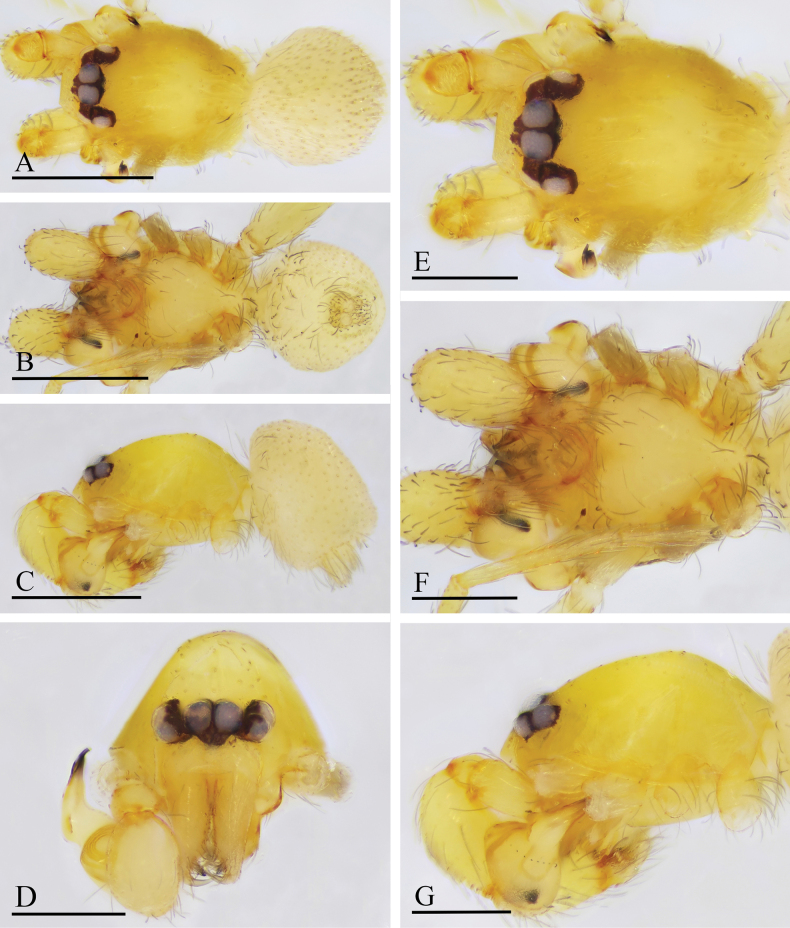
*Orchestina aureola* Tong & Li, 2011. A–C. Habitus, dorsal, ventral and lateral views; D–G. Prosoma, anterior, dorsal, ventral and lateral views. Scale bars: 0.4 mm (A–C); 0.2 mm (D–G).

**Figure 5.**
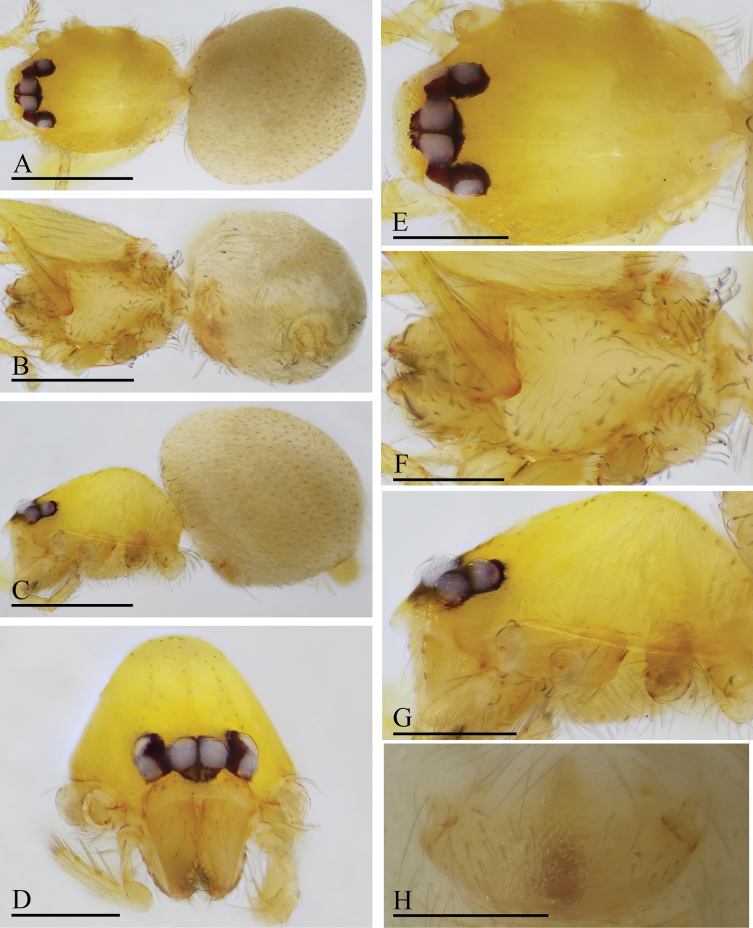
*Orchestina aureola* Tong & Li, 2011. A–C. Habitus, dorsal, ventral and lateral views; D–G. Prosoma, anterior, dorsal, ventral and lateral views; H. Epigaster, ventral view. Scale bars: 0.4 mm (A–C); 0.2 mm (D–H).

**Figure 6.**
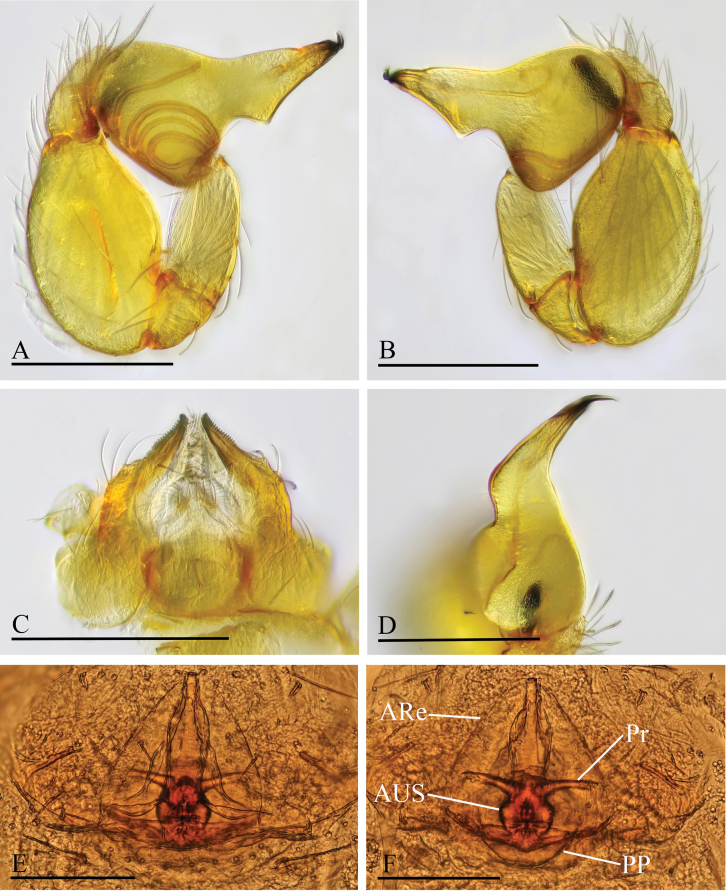
*Orchestina aureola* Tong & Li, 2011. A, B, D. Left palp, prolateral, retrolateral and dorsal views; C. Endites and labium, ventral view; E, F. Endogyne, ventral and dorsal views. Abbreviations: ARe = anterior receptaculum; AUS = anterior uterine sclerite; PP = posterior plate; Pr = protrusion. Scale bars: 0.2 mm (A, B, D); 0.1 mm (C, E, F).

##### Diagnosis and description.

See Tong and Li (2011).

##### Distribution.

China (Hainan, Yunnan).

#### ﻿ Orchestina caixiaae

Taxon classificationAnimalia

Tong & Li sp. nov.

BA8B70B3-1B96-5CB7-9576-0695B23AEF9F

https://zoobank.org/3641E473-6FD2-47E7-81DE-A53585D5413F

Figs 7 , 8 , 9

##### Type material.

***Holotype*** China • ♂ (SYNU-1588), fogging; Yunnan, Mengla Co., Menglun Town, XNNR, 55 Km., the secondary forest on the mountain top; 21°57.978'N, 101°12.167'E, 842 ± 14 m; 18.VIII.2011; Zheng G., Zhao Q. & Gao C. leg. ***Paratypes*.** China • 1 ♂ (SYNU-F-3956), same data as holotype • 1 ♀ (SYNU-F-4418), same data as holotype • 1 ♂ (SYNU-1589), fogging; XNNR, the pomelo forest beside rice field; 21°54.116'N, 101°16.15'E, 549 m; 18.VIII.2018; Bai Z., Yu H., Yang Y. & Chen Z. leg. • 1 ♂ (SYNU-1590), fogging; XNNR, 55 Km., the valley forest beside the artificial fishpond; 21°57.883'N, 101°12.147'E, 839 ± 18 m; 15.VIII.2011; Zheng G., Zhao Q. & Gao C. leg. • 4 ♀ (SYNU-F-4364–67), same data as above • 1 ♂ (SYNU-F-3957), same data as above.

##### Additional material.

China • 1 ♀ (SYNU-F-4359), fogging; Yunnan, Mengla Co., Xiaolongha Vill., Biodiversity corridor, valley forest; 21°24.253'N, 101°36.324'E, 761 ± 16 m; 15.VI.2013; Zhao Q. & Gao C. leg. • 1 ♀ (SYNU-F-4419), same data as above • 1 ♀ (SYNU-F-4356), fogging; XNNR, 55 Km., about 1 km up the hill, seasonal rainforest; 21°57.935'N, 101°12.305'E, 781 ± 17 m; 13.VIII.2011; Zheng G., Zhao Q. & Gao C. leg. • 1 ♀ (SYNU-F-4441), fogging; Xiaolongha Vill., Biodiversity corridor, seasonal rainforest; 21°24.26'N, 101°37.296'E, 653 ± 15 m; 27.VI.2012; Zhao Q. & Chen Z. leg. • 2 ♀ (SYNU-F-4728–29), same data as above • 1 ♀ (SYNU-F-4629), fogging; XNNR, 48 Km., artificial forest; 21°53.997'N, 101°16.957'E, 593 ± 18 m; 11.VIII.2011; Zheng G., Zhao Q. & Gao C. leg. • 2 ♀ (SYNU-F-4763–64), fogging; XNNR, 200 m east of the Lvshilin, limestone monsoon rainforest; 21°54.617'N, 101°16.843'E, 738 ± 17 m; 7.VIII.2011; Zheng G., Zhao Q. & Gao C. leg.

##### Diagnosis.

The new species is similar to *Orchestina lini* Tong & Li, 2025 in the non-enlarged palpal tibia and the abruptly bent sperm duct, but can be distinguished by the short tube-shaped embolus (vs very long; cf. Fig. 9A, B, D and Zhou et al. 2025: fig. 2A–C), the rectangular labium (vs rounded; cf. Fig. 9C and Zhou et al. 2025: fig. 1G), and the long medial clavate sclerite of endogyne (vs short; cf. Fig. 9F and Zhou et al. 2025: fig. 2F).

##### Description.

**Figure 7.**
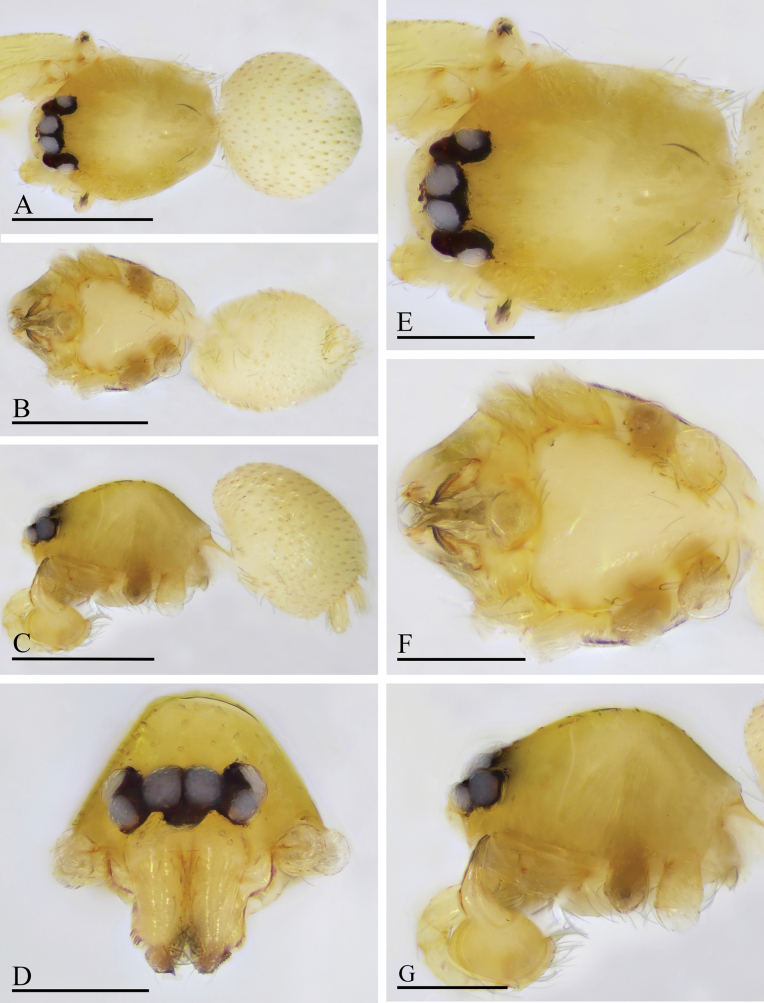
*Orchestina caixiaae* sp. nov., holotype male. A–C. Habitus, dorsal, ventral and lateral views; D–G. Prosoma, anterior, dorsal, ventral and lateral views. Scale bars: 0.4 mm (A–C); 0.2 mm (D–G).

**Male** (holotype). ***Body***: habitus as in Fig. 7A–C; body length 1.13. ***Carapace*** (Fig. 7E): 0.55 long, 0.41 wide. ***Eyes*** (Fig. 7D, E): well developed, PME largest. ***Clypeus*** as in Fig. 7D, G. ***Sternum*** as in Fig. 7F. ***Mouthparts*** (Figs 7D, F, 9C): labium rectangular, with anterior margin anteriorly projecting at middle, not fused to sternum; endites strongly sclerotized on outer margin, with serrula in single row, outer margin straight. ***Abdomen*** ovoid, 0.58 long; dorsum soft portions pale white, without color pattern. Legs: yellow, without color pattern; femur IV thickened, wider than femora I−III. ***Palp*** (Fig. 9A, B, D): tibia not enlarged, length/width = 2.04, cymbium elongated ovoid; bulb pear-shaped in lateral view, with ventral side strongly protruding proximally, ca 2.23× as wide as tibia; the sperm duct abruptly bent in the middle section, with two loops in prolateral view; embolus tapered, tube-shaped.

**Figure 8.**
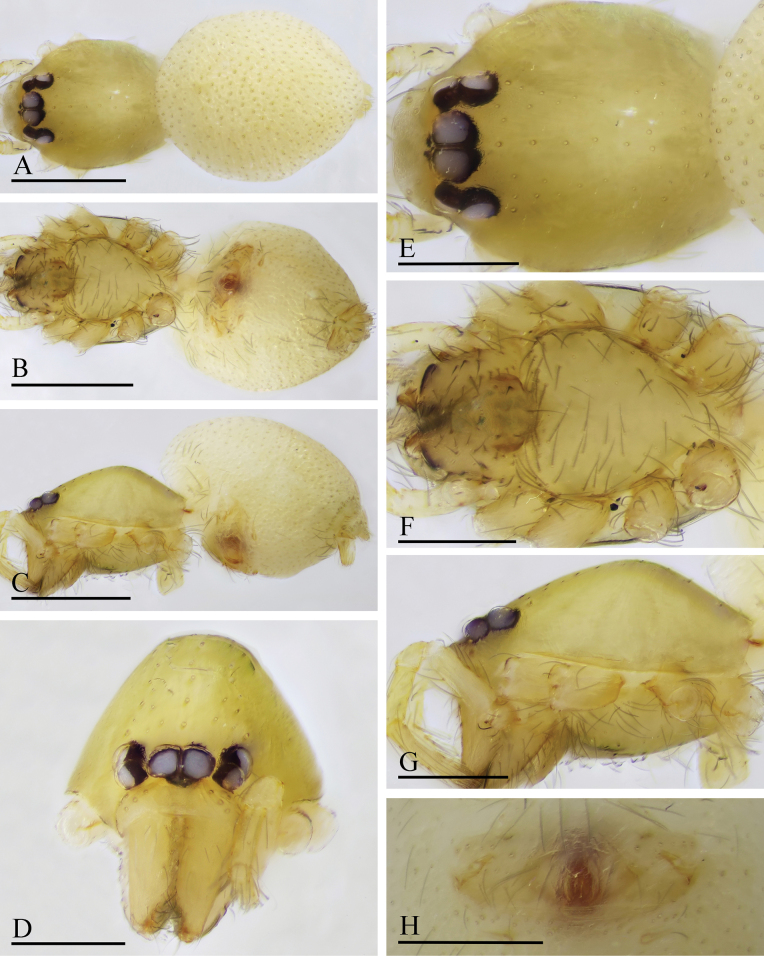
*Orchestina caixiaae* sp. nov., paratype female. A–C. Habitus, dorsal, ventral and lateral views; D–G. Prosoma, anterior, dorsal, ventral and lateral views; H. Epigaster, ventral view. Scale bars: 0.4 mm (A–C); 0.2 mm (D–H).

**Female** (SYNU-F-4418). Same as male except as noted. ***Body***: habitus as in Fig. 8A–C; body length 1.15. ***Carapace*** (Fig. 8E, G): 0.56 long, 0.45 wide. ***Abdomen***: 0.61 long. ***Epigaster*** (Fig. 8H): without special external features.

**Figure 9.**
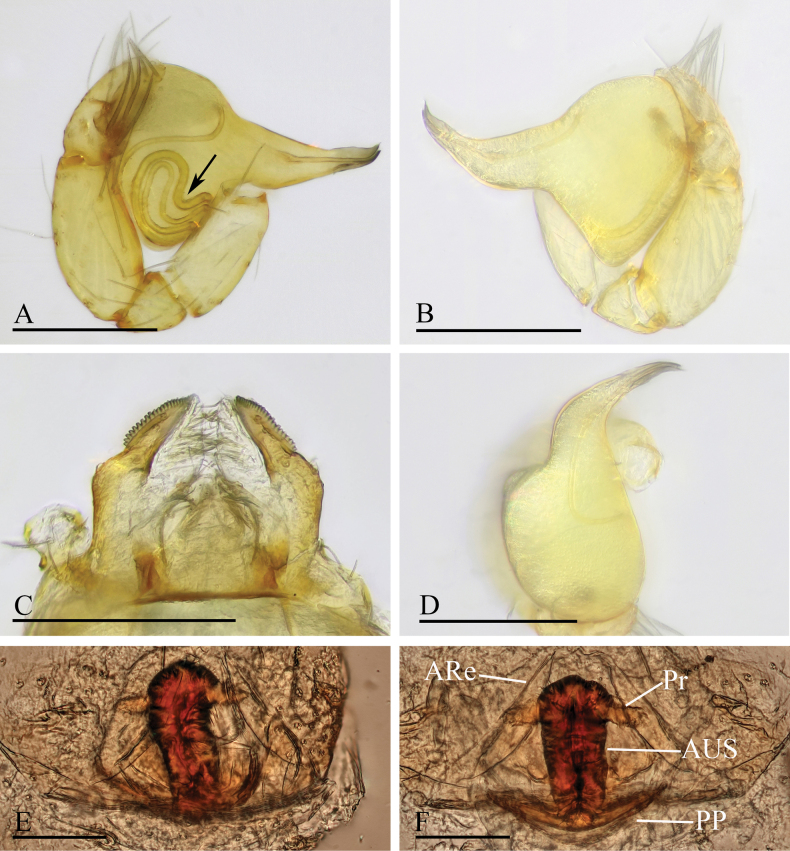
*Orchestina caixiaae* sp. nov. A, B, D. Left palp, prolateral, retrolateral and dorsal views, arrow in A. shows the abruptly bent; C. Endites and labium, ventral view; E, F. Endogyne, ventral and dorsal views. Abbreviations: ARe = anterior receptaculum; AUS = anterior uterine sclerite; PP = posterior plate; Pr = protrusion. Scale bars: 0.2 mm (A, B, D); 0.1 mm (C, E, F).

***Endogyne*** (Fig. 9E, F): with long medial clavate sclerite (AUS), provided with pair of short lateral protrusions (Pr); anterior receptaculum (ARe) triangular, semitransparent, slightly longer than AUS; posterior plate (PP) present, large.

##### Distribution.

Known only from the type locality.

##### Etymology.

The specific name is named after the collector, Caixia Gao.

#### ﻿ Orchestina longituba

Taxon classificationAnimalia

Tong & Li sp. nov.

B1D105CD-2D02-50BC-A4AC-2CEC76AD2992

https://zoobank.org/8d116056-8383-4d56-944a-db1d5762240d

Figs 10 , 11 , 12

##### Type material.

***Holotype*** China • ♂ (SYNU-1601), fogging; Yunnan, Mengla Co., Menglun Town, XNNR, 200 m east of the Lvshilin, limestone monsoon rainforest; 21°54.329'N, 101°16.715'E, 689 ± 19 m; 6.VIII.2011; Zheng G., Zhao Q. & Gao C. leg. ***Paratypes*.** China • 1 ♂ (SYNU-1602), same data as holotype • 2 ♂ (SYNU-F-4399–4400), same data as holotype • 15 ♀ (SYNU-F-4401–15); same data as holotype • 3 ♀ (SYNU-1568–70), fogging; XNNR, 48 Km.; 21°58.704'N, 101°19.748'E, 1088 ± 12 m; 12.VIII.2011; Zhao Q. & Chen Z. leg. • 4 ♀ (SYNU-1571–74), fogging; Nanshahe Vill., seasonal rainforest; 21°36.201'N, 101°34.702'E, 826 ± 43 m; 14.VII.2012; Zhao Q. & Chen Z. leg.

##### Additional material.

China • 1 ♂ 1 ♀ (SYNU-F-3953–54), same data as holotype • 3 ♂ (SYNU-F-4147–49), same data as holotype • 8 ♀ (SYNU-F-4597–4604), same data as holotype • 2 ♀ (SYNU-F-4594–95), same data as holotype • 1 ♂ (SYNU-1603), fogging; XTBG, Ficus microcarpa forest; 21°34.284'N, 101°15.986'E, 538 ± 9 m; 22.VIII.2011; Zheng G., Zhao Q. & Gao C. leg. • 7 ♂ (SYNU-F-4092–98), fogging; Mengyang Town, seasonal rainforest; 22°09.765'N, 100°52.553'E, 862 ± 33 m; 22.VII.2012; Zhao Q. & Chen Z. leg. • 11 ♀ (SYNU-F-4583–93), same data as above • 6 ♂ (SYNU-F-4184–89), same data as above • 13 ♀ (SYNU-F-4566–78), same data as above • 6 ♀ (SYNU-F-4429–34), fogging; XNNR, 48 Km.; 21°58.704'N, 101°19.748'E, 1088 ± 12 m; 12.VIII.2011; Zhao Q. & Chen Z. leg. • 6 ♀ (SYNU-F-4491–96), same data as above • 1 ♂ (SYNU-F-4180), same data as above • 1 ♂ 2 ♀ (SYNU-F-4554–56), same data as above • 4 ♀ (SYNU-F-4562–65), same data as above • 1 ♀ (SYNU-F-4742), same data as above • 1 ♂ (SYNU-F-4181), fogging; XNNR, 200 m east of the Lvshilin, limestone monsoon rainforest; 21°54.617'N, 101°16.843'E, 738 ± 17 m; 7.VIII.2011; Zheng G., Zhao Q. & Gao C. leg. • 23 ♀ (SYNU-F-4376–98), same data as above • 1 ♂ (SYNU-F-3955), fogging; XNNR, 55 Km., the valley forest beside the artificial fishpond; 21°57.883'N, 101°12.147'E, 839 ± 18 m; 15.VIII.2011; Zheng G., Zhao Q. & Gao C. leg. • 5 ♀ (SYNU-F-4436–40), same data as above • 32 ♂ (SYNU-F-4104–35), same data as above • 45 ♀ (SYNU-F-4509–53), same data as above • 5 ♂ (SYNU-F-4099–4103), fogging; XNNR, 55 Km., about 1 km up the hill, seasonal rainforest; 21°57.949'N, 101°12.247'E, 788 ±20 m; 13.VIII.2011; Zheng G., Zhao Q. & Gao C. leg. • 20 ♀ (SYNU-F-4471–90), same data as above • 2 ♂ (SYNU-F-4175–76), fogging; XNNR, 55 Km., secondary forest; 21°57.978'N, 101°12.167'E, 842 ± 14 m; 18.VIII.2011; Zheng G., Zhao Q. & Gao C. leg. • 1 ♀ (SYNU-F-4851), same data as above • 6 ♀ (SYNU-F-4722–27), same data as above • 1 ♂ 2 ♀ (SYNU-F-4620–22), fogging; XNNR, 55 Km., about 1 km up the hill, seasonal rainforest; 21°57.935'N, 101°12.305'E, 781 ± 17 m; 13.VIII.2011; Zheng G., Zhao Q. & Gao C. leg. • 9 ♀ (SYNU-F-4605–13), same data as above • 1 ♀ (SYNU-F-4738), fogging; XNNR, 55 Km., seasonal rainforest; 21°57.531'N, 101°11.961'E, 751 ± 17 m; 13.VIII.2011; Zhao Q. & Chen Z. leg. • 1 ♀ (SYNU-F-4850), fogging; XNNR, 55 Km., beside the one-hectare-sample-plot of Ecological Protection Station, valley forest; 21°57.010'N, 101°12.058'E, 814 ± 22 m; 8.VIII.2011; Zheng G., Zhao Q. & Gao C. leg. • 2 ♂ (SYNU-F-4090–91), fogging; Nanshahe Vill., seasonal rainforest; 21°36.388'N, 101°34.246'E, 797 ± 35 m; 13.VII.2012; Zhao Q. & Chen Z. leg. • 1 ♀ (SYNU-F-4416), same data as above • 10 ♀ (SYNU-F-4746–55), same data as above • 3 ♂ (SYNU-F-4136–38), fogging; Jinghong city, Meng’a Town, Wengnan Vill., secondary forest; 22°04'59.8"N, 100°22'13.4"E, 1137 ± 12 m; 25.VII.2012; Zhao Q. & Chen Z. leg. • 1 ♂ 4 ♀ (SYNU-F-4557–61), same data as above • 2 ♀ (SYNU-F-4623–24), same data as above • 4 ♂ (SYNU-F-4139–42), fogging; Huigang Vill., Xilu habitat restoration area, monsoon forest; 21°37.045'N, 101°35.268'E, 764 ± 25 m; 12.VII.2012; Zhao Q. & Chen Z. leg. • 6 ♂ (SYNU-F-4153–58), same data as above • 15 ♂ (SYNU-F-4159–73), same data as above • 3 ♂ (SYNU-F-4177–79), same data as above • 8 ♀ (SYNU-F-4370), same data as above • 9 ♀ (SYNU-F-4420–28), same data as above • 12 ♀ (SYNU-F-4497–4508), same data as above • 4 ♂ (SYNU-F-4143–46), fogging; Xiaolongha Vill., Biodiversity corridor, seasonal rainforest; 21°24.798'N, 101°37.88'E, 693 ± 46 m; 28.VI.2012; Zhao Q. & Chen Z. leg. • 6 ♀ (SYNU-F-4614–19), same data as above • 1 ♂ (SYNU-F-4152), same data as above • 1 ♂ (SYNU-F-4183), same data as above • 4 ♂ (SYNU-F-4190–93), fogging; Xiaolongha Vill., Biodiversity corridor, seasonal rainforest; 21°24.265'N, 101°37.296'E, 653 ± 15 m; 27.VI.2012; Zhao Q. & Chen Z. leg. • 1 ♀ (SYNU-F-4196), same data as above • 1 ♀ (SYNU-F-4730), same data as above • 23 ♀ (SYNU-F-4441–63), same data as above • 7 ♀ (SYNU-F-4728–4734), same data as above • 1 ♂ 1 ♀ (SYNU-F-4194–95), fogging; Xiaolongha Vill., Biodiversity corridor, seasonal rainforest; 21°24.23'N, 101°36.261'E, 715 ± 27 m; 4.VII.2012; Zhao Q. & Chen Z. leg. • 8 ♀ (SYNU-F-4686–93), same data as above • 1 ♀ (SYNU-F-4685), fogging; Xiaolongha Vill., Biodiversity corridor, seasonal rainforest; 21°24.213'N, 101°36.995'E, 834 ± 15 m; 3.VII.2012; Zhao Q. & Chen Z. leg. • 2 ♀ (SYNU-F-4744–45), same data as above • 1 ♀ (SYNU-F-4737), fogging; Xiaolongha Vill., Biodiversity corridor, valley forest; 21°24.253'N, 101°36.324'E, 761 ± 16 m; 15.VI.2013; Zhao Q. & Chen Z. leg. • 1 ♀ (SYNU-F-4743), fogging; Xiaolongha Vill., Biodiversity corridor, secondary rainforest; 21°24.33'N, 101°37.021'E, 801 ± 22 m; 23.VI.2012; Zhao Q. & Chen Z. leg. • 2 ♀ (SYNU-F-4739–40), same data as above • 1 ♂ (SYNU-F-4174), fogging; XTBG, site 2, by the roadside; 21°53.748'N, 101°17.084'E, 619 m; 1.V.2019; Bai Z., Yu H., Wang C., Tong Y. & Chen Z. leg. • 1 ♂ (SYNU-F-4182), fogging; Jinghong City, Mandazhai Vill., secondary rainforest; 22°01.702'N, 100°23.696'E, 1188 ± 18 m; 28.VII.2012; Zhao Q. & Chen Z. leg. • 4 ♀ (SYNU-F-4579–82), same data as above • 8 ♀ (SYNU-F-4698–4705), fogging; Jinghong City, Guanping Town, seasonal rainforest; 21°13.353'N, 100°53.251'E, 832 ± 12 m; 21.VII.2012; Zhao Q. & Chen Z. leg. • 1 ♂ 4 ♀ (SYNU-F-4676–80), same data as above • 7 ♀ (SYNU-F-4464–70), fogging; XNNR, 48 Km., artificial forest; 21°53.997'N, 101°16.957'E, 593 ± 18 m; 11.VIII.2011; Zheng G., Zhao Q. & Gao C. leg. • 3 ♀ (SYNU-F-4625–27), fogging; XNNR, 55 Km., valley forest; 21°58.610'N, 101°12.058'E, 814 ± 22 m; 17.VIII.2011; Zheng G., Zhao Q. & Gao C. leg. • 1 ♀ (SYNU-F-4628), fogging; XNNR, 48 Km.; 21°58.704'N, 101°19.748'E, 1088 ± 12 m; 12.VIII.2011; Zhao Q. & Chen Z. leg. • 2 ♀ (SYNU-4630–31), same data as above • 1 ♀ (SYNU-F-4632), same data as above • 1 ♀ (SYNU-F-4852), same data as above • 22 ♀ (SYNU-F-4644–65), fogging; XNNR, 48 Km.; 21°58.764'N, 101°09.748'E, 1038 ± 12 m; 10.VIII.2011; Zhao Q. & Chen Z. leg. • 4 ♀ (SYNU-F-4668–71), same data as above • 1 ♂ 3 ♀ (SYNU-F-4672–75), fogging; XNNR, 48 Km., seasonal rainforest; 21°38.853'N, 101°09.625'E, 1001 ± 19 m; 30.VII.2011; Zheng G., Zhao Q. & Gao C. leg. • 1 ♂ 7 ♀ (SYNU-F-4633–40), fogging; XTBG, Ficus microcarpa forest; 21°54.565'N, 101°16.021'E, 584 ± 14 m; 21.VIII.2011; Zheng G., Zhao Q. & Gao C. leg. • 1 ♀ (SYNU-F-4757), fogging; XTBG, site 5, 21°53.616'N, 101°18.250'E, 523 m; 29.IV.2019; Bai Z., Yu H., Wang C., Tong Y. & Chen Z. leg. • 1 ♀ (SYNU-F-4758), fogging; XTBG, site 7, 21°54.337'N, 101°16.793'E, 618 m; 2.V.2019; Bai Z., Yu H., Wang C., Tong Y. & Chen Z. leg. • 2 ♀ (SYNU-F-4735–36), fogging; Jinghong City, Guanping Town, seasonal rainforest; 22°13.681'N, 100°53.363'E, 888 ± 18 m; 20.VII.2012; Zhao Q. & Chen Z. leg.

##### Diagnosis.

The new species is similar to *Orchestina codalmasi* Wunderlich, 2011 (female unknown) and *Orchestina sublongituba* sp. nov. in the strongly coiled sperm duct and the enlarged palpal tibia, but can be distinguished from *O. codalmasi* by the very long tube-shaped embolus (vs short; cf. Fig. 12A, B, D and Wunderlich 2008: fig. 7); from *O. sublongituba* by palpal tibia narrower than bulbus (vs wider; cf. Fig. 12A, B and Fig. 21A, B), the diamond-shaped labium (vs tongue-shaped; cf. Fig. 12C and Fig. 21C) and the obscure marking on epigaster (vs saddle-shaped; cf. Fig. 11H and Fig. 20H).

##### Description.

**Figure 10.**
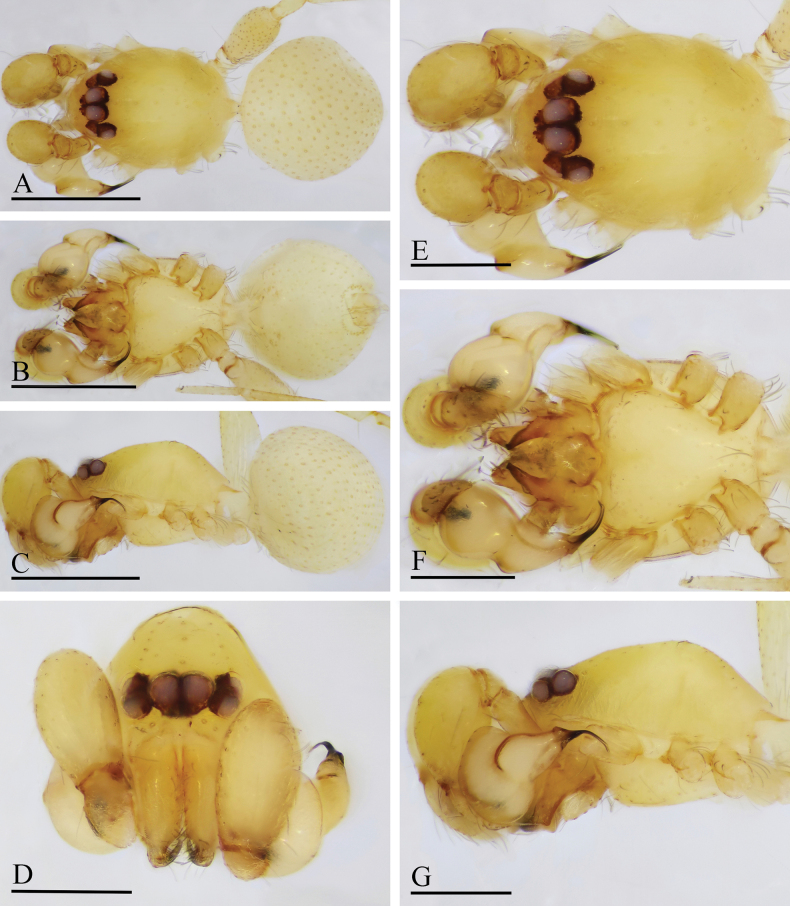
*Orchestina longituba* sp. nov., holotype male. A–C. Habitus, dorsal, ventral and lateral views; D–G. Prosoma, anterior, dorsal, ventral and lateral views. Scale bars: 0.4 mm (A–C); 0.2 mm (D–G).

**Male** (holotype). ***Body***: habitus as in Fig. 10A–C; body length 1.25. ***Carapace*** (Fig. 10E, G): 0.54 long, 0.39 wide; yellow, oval in dorsal view. ***Eyes*** (Fig. 10D, E): well developed, PME largest. ***Clypeus*** as in Fig. 10D. ***Sternum*** as in Fig. 10F. ***Mouthparts*** (Figs 10D, F, 12C): labium diamond-shaped, not fused to sternum, anterior margin not indented at middle; endites strongly sclerotized, with serrula in single row, out margin straight. ***Abdomen*** ovoid, 0.58 long; dorsum soft portions pale white, without color pattern. ***Legs***: yellow, without color pattern; femur IV thickened, wider than femora I−III. ***Palp*** (Fig. 12A, B, D): tibia strongly enlarged, length/width = 1.61, cymbium elongated ovoid; bulb pear-shaped in lateral view, with ventral side strongly protruding proximally, ca 1.25× as wide as tibia; the sperm duct with two loops in prolateral view; embolus long tube-shaped, with a basal node (Fig. 12A, B).

**Figure 11.**
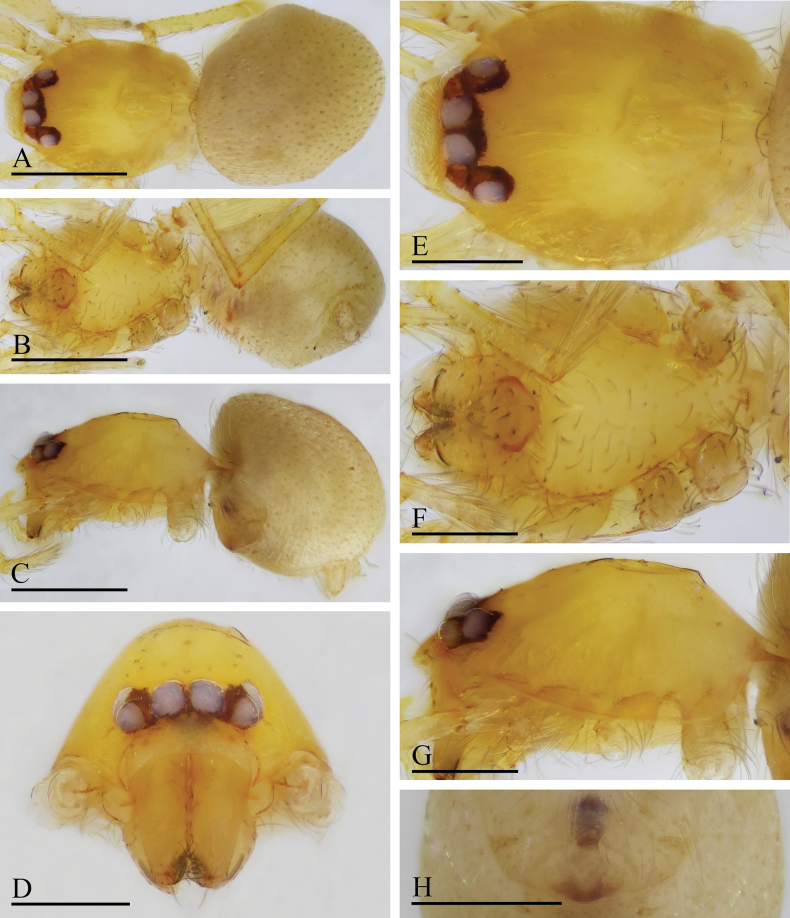
*Orchestina longituba* sp. nov., paratype female. A–C. Habitus, dorsal, ventral and lateral views; D–G. Prosoma, anterior, dorsal, ventral and lateral views; H. Epigaster, ventral view. Scale bars: 0.4 mm (A–C); 0.2 mm (D–H).

**Female** (SYNU-1568). Same as male except as noted. ***Body***: habitus as in Fig. 11A–C; body length 1.28. ***Carapace*** (Fig. 11E, G): 0.67 long, 0.48 wide. ***Abdomen***: 0.61 long. ***Epigaster*** (Fig. 11H): without special external features.

**Figure 12.**
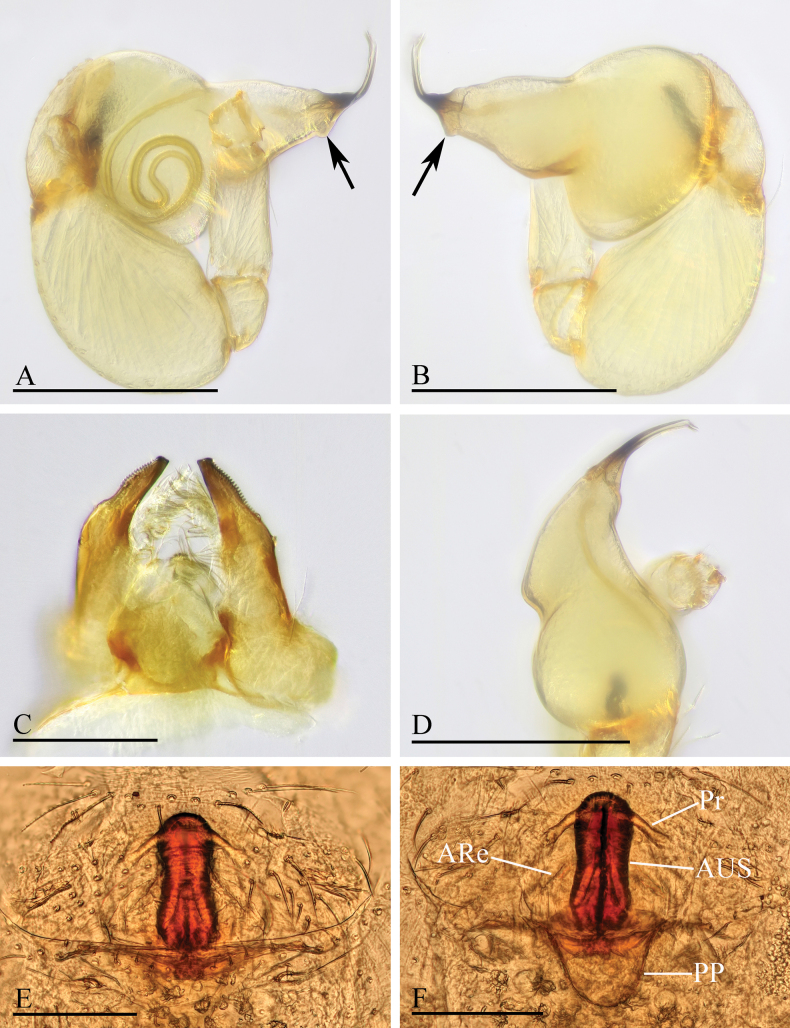
*Orchestina longituba* sp. nov. A, B, D. Left palp, prolateral, retrolateral and dorsal views, arrows in A, B. show the basal node; C. Endites and labium, ventral view; E, F. Endogyne, ventral and dorsal views. Abbreviations: ARe = anterior receptaculum; AUS = anterior uterine sclerite; PP = posterior plate; Pr = protrusion. Scale bars: 0.2 mm (A, B, D); 0.1 mm (C, E, F).

***Endogyne*** (Fig. 12E, F): with stout medial clavate sclerite (AUS), provided with pair of short lateral protrusions (Pr); lateral protrusions at angle of about 60° with medial clavate sclerite; anterior receptaculum (ARe) triangular, semitransparent, shorter than AUS; posterior plate (PP) present, large.

##### Distribution.

Known only from the type locality.

##### Etymology.

The specific name is derived from the Latin and refers to the long tube-shaped distal end of bulb.

#### ﻿ Orchestina qingyuani

Taxon classificationAnimalia

Tong & Li sp. nov.

2D3BB9F1-B4ED-565A-9EEA-99E6A4C359A4

https://zoobank.org/06A73274-99B9-4CDF-B60C-7558B4E20C03

Figs 13 , 14 , 15

##### Type material.

***Holotype*** China • ♂ (SYNU-1583), fogging; Yunnan, Mengla Co., Menglun Town, XNNR, 55 Km., valley forest; 21°58.610'N, 101°12.058'E, 814 ± 22 m; 17.VIII.2011; Zheng G., Zhao Q. & Gao C. leg. ***Paratypes*.** China • 2 ♂ (SYNU-1584–85), fogging; XNNR, 55 Km., the valley forest beside the artificial fishpond; 21°57.883'N, 101°12.147'E, 839 ± 18 m; 15.VIII.2011; Zheng G., Zhao Q. & Gao C. leg. • 4 ♂ (SYNU-F-3932–35), same data as above • 5 ♂ (SYNU-F-3948–52), same data as above • 2 ♀ (SYNU-F-4362–4363), same data as above • 1 ♀(SYNU-1562), fogging; XNNR, 48 Km.; 21°58.764'N, 101°09.748'E, 1038 ± 12 m; 10.VIII.2011; Zheng G., Zhao Q. & Gao C. leg. • 1 ♀ (SYNU-F-4643), same data as above • 1 ♀ (SYNU-F-4353), same data as above • 1 ♀ (SYNU-F-4354), same data as above.

##### Additional material.

China • 2 ♂ (SYNU-1586–87), fogging; Xiaolongha Vill., Biodiversity corridor, seasonal rainforest; 21°24.265'N, 101°37.296'E, 653 ± 15 m; 27.VI.2012; Zhao Q. & Chen Z. leg. • 1 ♂ (SYNU-F-3939), same data as above • 2 ♀ (SYNU-F-4761–62), fogging; XNNR, 55 Km., about 1 km up the hill, seasonal rainforest; 21°57.935'N, 101°12.305'E, 781 ± 17 m; 13.VIII.2011; Zheng G., Zhao Q. & Gao C. leg. • 1 ♂ (SYNU-F-3938), fogging; Bubang Town; 21°36.640'N, 101°34.905'E, 823 ± 35 m; 10.VII.2011; Zhao Q. & Chen Z. leg. • 2 ♂ (SYNU-F-3946–47), fogging; Xiaolongha Vill., Biodiversity corridor, seasonal rainforest; 21°24.798'N, 101°37.880'E, 693 ± 46 m; 28.VI.2012; Zhao Q. & Chen Z. leg. • 2 ♀ (SYNU-F-4357–58), fogging; Xiaolongha Vill., Biodiversity corridor, valley forest; 21°24.253'N, 101°36.324'E, 761 ± 16 m; 15.VI.2013; Zhao Q. & Chen Z. leg. • 1 ♂ (SYNU-F-3940), fogging; XNNR, 55 Km., about 1 km up the hill, seasonal rainforest; 21°57.949'N, 101°12.247'E, 788 ± 20 m; 13.VIII.2011; Zheng G., Zhao Q. & Gao C. leg. • 1 ♀ (SYNU-F-4760), same data as above • 1 ♀ (SYNU-F-4846), fogging; XNNR, 55 Km., the valley forest beside the artificial fishpond; 21°57.883'N, 101°12.147'E, 839 ± 18 m; 15.VIII.2011; Zheng G., Zhao Q. & Gao C. leg. • 1 ♂ (SYNU-F-3942), fogging; Huigang Vill., Xilu habitat restoration area, monsoon forest; 21°37.045'N, 101°35.268'E, 764 ± 25 m; 12.VII.2012; Zhao Q. & Chen Z. leg. • 1 ♀ (SYNU-F-4360), same data as above • 1 ♂ (SYNU-F-3943), fogging; XNNR, 200 m east of the Lvshilin, limestone monsoon rainforest; 21°54.617'N, 101°16.843'E, 738 ± 17 m; 7.VIII.2011; Zheng G., Zhao Q. & Gao C. leg. • 1 ♂ 1 ♀ (SYNU-F-4197–98), same data as above • 2 ♂ (SYNU-F-3944–45), fogging; XNNR, 200 m east of the Lvshilin, limestone monsoon rainforest; 21°54.329'N, 101°16.715'E, 689 ± 19 m; 6.VIII.2011; Zheng G., Zhao Q. & Gao C. leg.

##### Diagnosis.

The new species is similar to *Orchestina flava* Ono, 2005 in the yellow carapace and the strongly coiled sperm duct, but can be distinguished by the strongly enlarged palpal tibia (vs not enlarged; cf. Fig. 15A, B and Ono 2005: figs 8–10), the rectangular labium (vs rounded; cf. Fig. 15C and Ono 2005: fig. 3) and the obscure marking of endogyne (vs nearly triangular marking; cf. Fig. 14H and Ono 2005: fig. 6).

##### Description.

**Figure 13.**
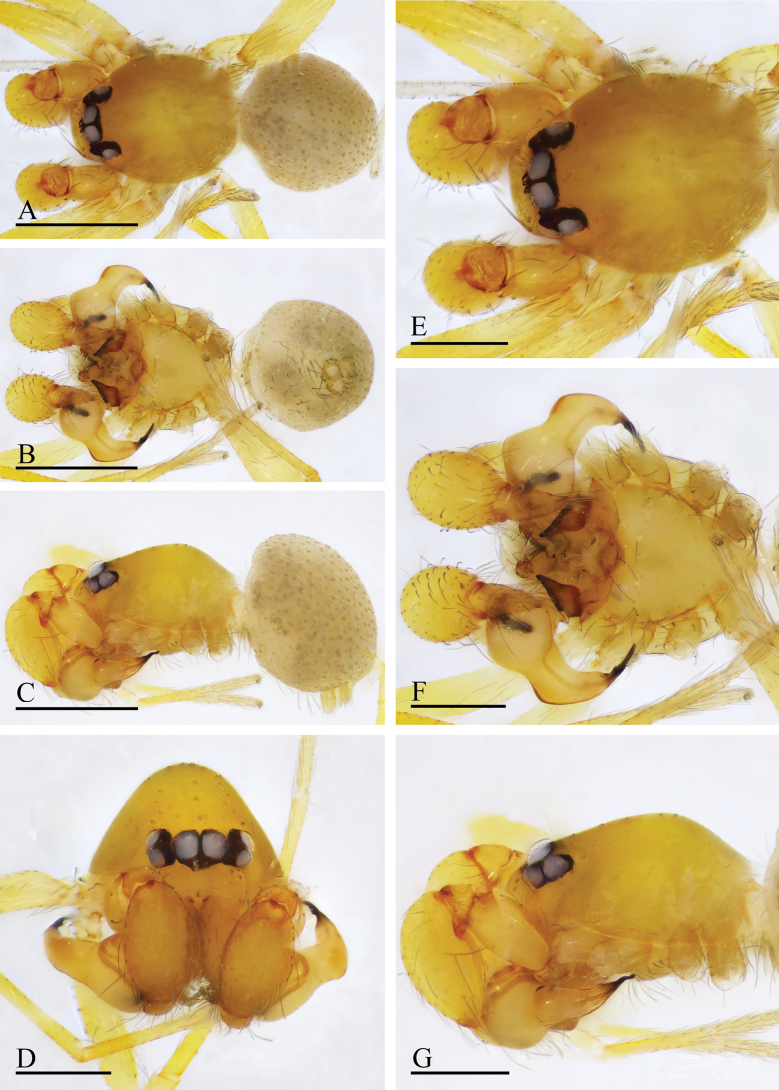
*Orchestina qingyuani* sp. nov., holotype male. A–C. Habitus, dorsal, ventral and lateral views; D–G. Prosoma, anterior, dorsal, ventral and lateral views. Scale bars: 0.4 mm (A–C); 0.2 mm (D–G).

**Male** (holotype). ***Body***: habitus as in Fig. 13A–C; body length 1.05. ***Carapace*** (Fig. 13E, G): 0.54 long, 0.42 wide; yellow. ***Eyes*** (Fig. 13D, E): well developed, PME largest. ***Clypeus*** as in Fig. 13D. ***Sternum*** as in Fig. 13F. ***Mouthparts*** (Figs 13D, F, 15C): labium rectangular, with anterior margin anteriorly projecting at middle, not fused to sternum; endites strongly sclerotized, with serrula in single row, outer margin smoothly curved. ***Abdomen*** ovoid, 0.48 long; dorsum soft portions pale white, without color pattern. ***Legs***: yellow, without color pattern; femur IV thickened, wider than femora I−III. ***Palp*** (Fig. 15A, B, D): tibia strongly enlarged, length/width = 1.87, cymbium small; bulb long pear-shaped in lateral view, with ventral side strongly protruding proximally, ca 1.09× as wide as tibia; the sperm duct with two loops in prolateral view; embolus long tube-shaped, with a sub-apical node.

**Figure 14.**
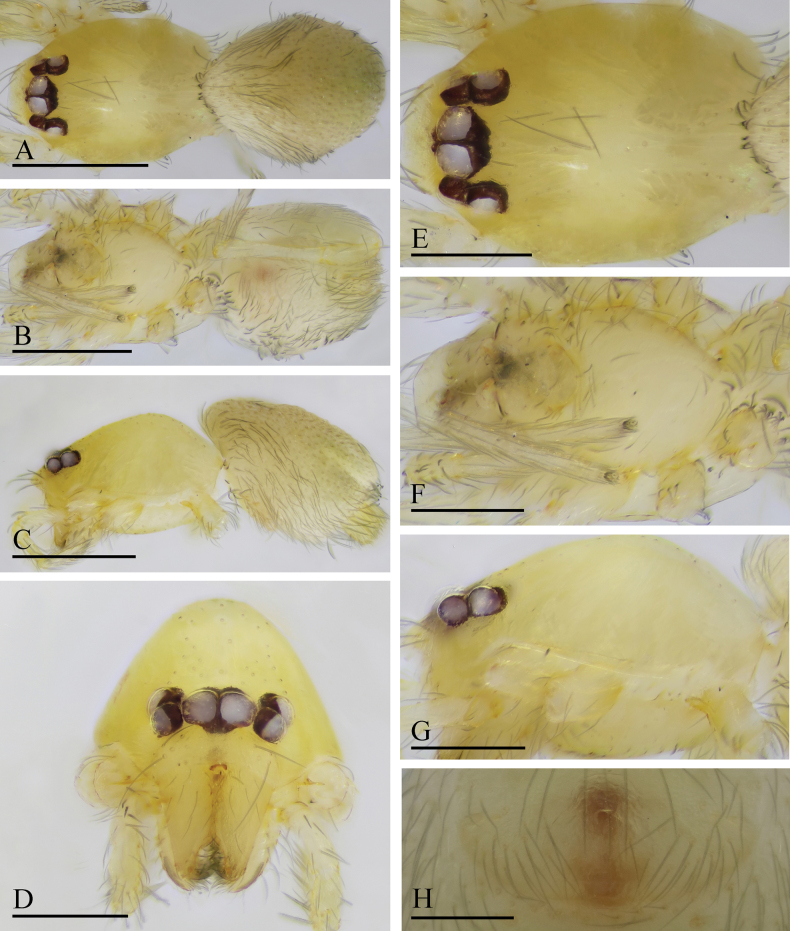
*Orchestina qingyuani* sp. nov., paratype female. A–C. Habitus, dorsal, ventral and lateral views; D–G. Prosoma, anterior, dorsal, ventral and lateral views; H. Epigaster, ventral view. Scale bars: 0.4 mm (A–C); 0.2 mm (D–G); 0.1mm (H).

**Female** (SYNU-1562). Same as male except as noted. ***Body***: habitus as in Fig. 14A–C; body length 1.11. ***Carapace*** (Fig. 14E, G): 0.62 long, 0.43 wide. ***Abdomen***: 0.51 long. ***Epigaster*** (Fig. 14H): without special external features.

**Figure 15.**
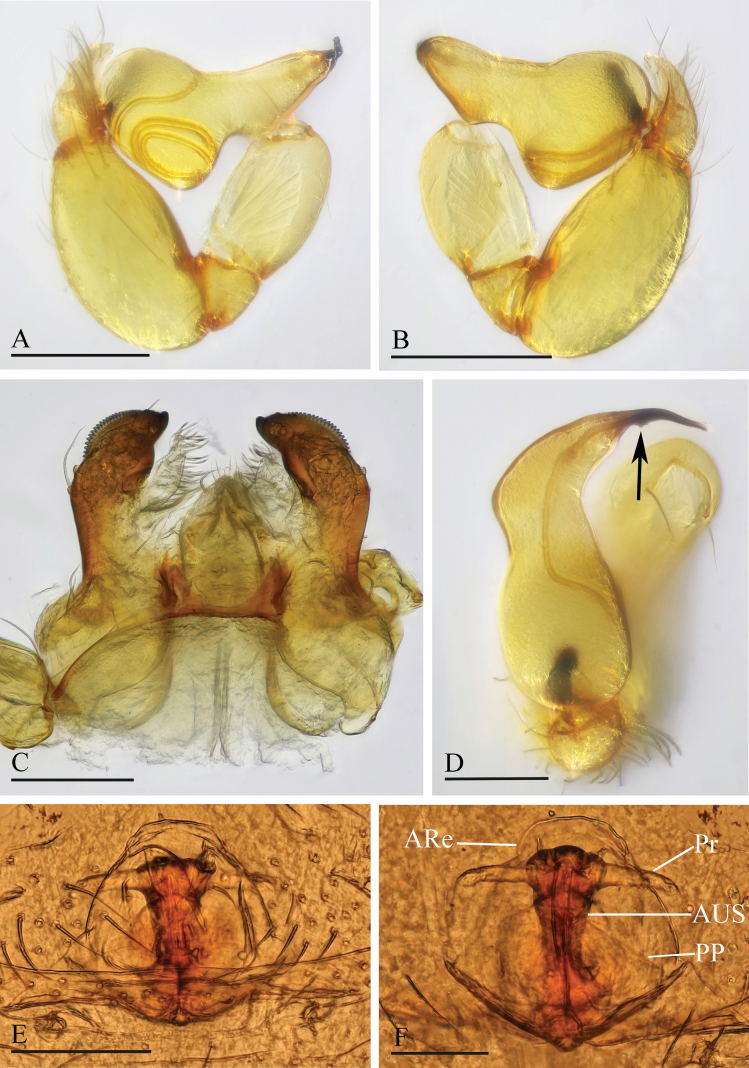
*Orchestina qingyuani* sp. nov. A, B, D. Left palp, prolateral, retrolateral and dorsal views, arrow in D. shows the node; C. Endites and labium, ventral view; E, F. Endogyne, ventral and dorsal views. Abbreviations: ARe = anterior receptaculum; AUS = anterior uterine sclerite; PP = posterior plate; Pr = protrusion. Scale bars: 0.2 mm (A, B); 0.1 mm (C–F).

***Endogyne*** (Fig. 15E, F): with slender medial clavate sclerite (AUS), provided with pair of long lateral protrusions (Pr); anterior receptaculum (ARe) rounded, semitransparent, slightly longer than AUS; posterior plate (PP) present, large.

##### Distribution.

Known only from the type locality.

##### Etymology.

The specific name is named after the collector, Qingyuan Zhao.

#### ﻿ Orchestina subconcava

Taxon classificationAnimalia

Tong & Li sp. nov.

844F07A3-7846-535D-AAB8-FC8D19ABA41B

https://zoobank.org/FF1D10AF-C7AA-4C57-B248-B989FDF63018

Figs 16 , 17 , 18

##### Type material.

***Holotype*** China • ♂ (SYNU-1575), fogging; Yunnan, Jinghong City, Mengyang Town, seasonal rainforest; 22°09.765'N, 100°52.553'E, 862 ± 33 m; 22.VII.2012; Zhao Q. & Chen Z. leg. ***Paratypes*.** China • 3 ♂ (SYNU-1576–78), same data as holotype • 10 ♂ (SYNU-3705–14), same data as holotype • 3 ♀(SYNU-1548–50), same data as holotype • 2 ♀ (SYNU-1551–52), same data as holotype • 14 ♀ (SYNU-F-4239–52), same data as holotype.

##### Additional material.

China • 17 ♂ (SYNU-F-3719–35), same data as holotype • 3 ♀ (SYNU-F-4211–13), same data as holotype • 25 ♀ (SYNU-F-4214–38), same data as holotype • 2 ♂ (SYNU-F-3703–04), fogging; XTBG, Ficus microcarpa forest; 21°34.284'N, 101°15.986'E, 538 ± 9 m; 22.VIII.2011; Zheng G., Zhao Q. & Gao C. leg. • 1 ♀ (SYNU-F-4257), same data as above • 1 ♂ (SYNU-F-3736), fogging; XTBG, 300 acres of riverbank, 13.V.2019; Bai Z., Chen Z., Wang C. & Yu H. leg. • 1 ♂ (SYNU-F-3737), fogging; Meng’a Town, Wengnan Vill., secondary forest; 22°05.020'N, 100°22.086'E, 1118 ± 10 m; 24.VII.2012; Zhao Q. & Chen Z. leg. • 1 ♀ (SYNU-F-4258), same data as above • 1 ♂ (SYNU-F-3738), fogging; XNNR, 200 m east of the Lvshilin, limestone monsoon rainforest; 21°54.617'N, 101°16.843'E, 738 ± 17 m; 7.VIII.2011; Zheng G., Zhao Q. & Gao C. leg. • 1 ♂ (SYNU-F-3739), fogging; XNNR, 55 Km., the valley forest beside the artificial fishpond; 21°57.883'N, 101°12.147'E, 839 ± 18 m; 15.VIII.2011; Zheng G., Zhao Q. & Gao C. leg. • 2 ♀ (SYNU-F-4202–03), fogging; Jinghong City, Guanping Town, seasonal rainforest; 21°13.353'N, 100°53.252'E, 832 ± 12 m; 21.VII.2012; Zhao Q. & Chen Z. leg. • 3 ♀ (SYNU-F-4207–09), same data as above • 3 ♀ (SYNU-F-4204–06), fogging; Xiaolongha Vill., Biodiversity corridor, seasonal rainforest; 21°24.265'N, 101°37.296'E, 653 ± 15 m; 27.VI.2012; Zhao Q. & Chen Z. leg. • 4 ♀ (SYNU-F-4259–62), same data as above • 2 ♀ (SYNU-F-4263–64), fogging; Xiaolongha Vill., Biodiversity corridor, seasonal rainforest; 21°24.213'N, 101°36.995'E, 834 ± 15 m; 3.VII.2012; Zhao Q. & Chen Z. leg. • 1 ♀ (SYNU-F-4741), fogging; Xiaolongha Vill., Biodiversity corridor, secondary rainforest; 21°24.330'N, 101°37.021'E, 801 ± 22 m; 23.VI.2012; Zhao Q. & Chen Z. leg. • 1 ♀ (SYNU-F-4210), fogging; Huigang Vill., Xilu habitat restoration area, monsoon forest; 21°37.045'N, 101°35.268'E, 764 ± 25 m; 12.VII.2012; Zhao Q. & Chen Z. leg. •1 ♀ (SYNU-F-4265), same data as above.

##### Diagnosis.

**Figure 16.**
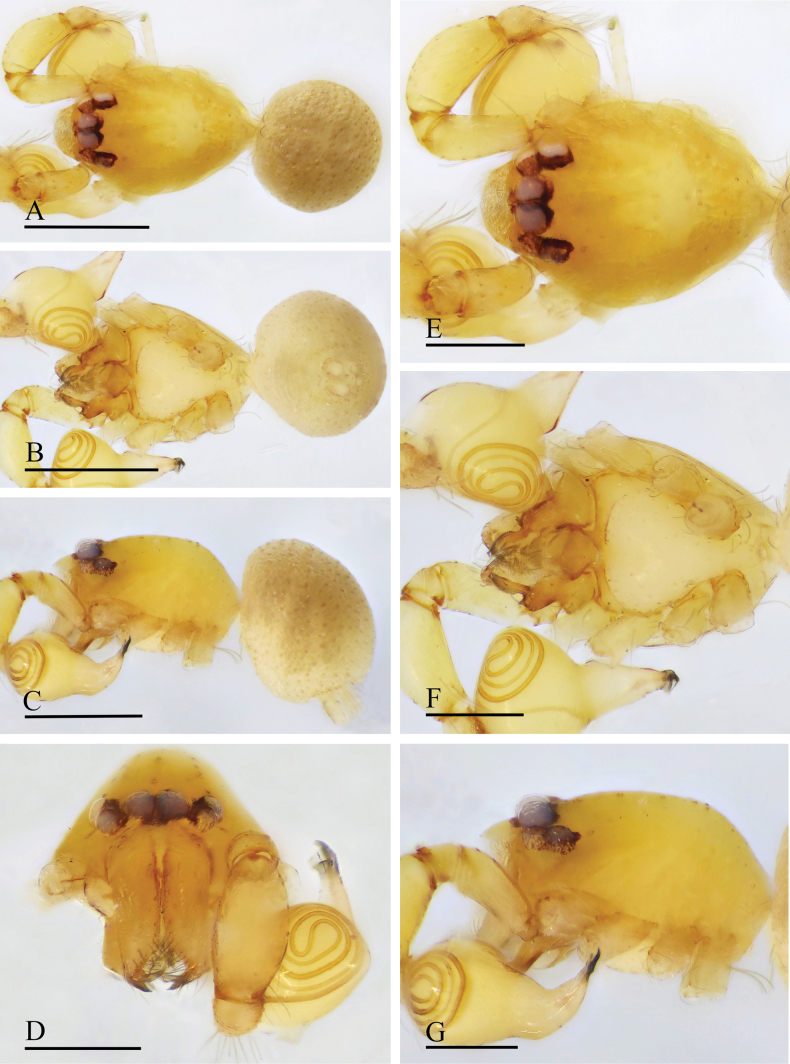
*Orchestina subconcava* sp. nov., holotype male. A–C. Habitus, dorsal, ventral and lateral views; D–G. Prosoma, anterior, dorsal, ventral and lateral views. Scale bars: 0.4 mm (A–C); 0.2 mm (D–G).

The new species is similar to *O. concava* Tong & Li, 2024 in the deeply excavated male endites, but can be distinguished by the absence of net-shaped pattern on carapace (vs with pattern; cf. Fig. 16A, E and Song et al. 2024: fig. 4A, D), the palpal tibia not enlarged (vs strongly enlarged; cf. Fig. 18A, B and Song et al. 2024: fig. 5A, B) and the sperm duct with three loops in prolateral view (vs two loops; cf. Fig. 18A and Song et al. 2024: fig. 5A).

##### Description.

**Male** (holotype). ***Body***: habitus as in Fig. 16A–C; body length 1.12. ***Carapace*** (Fig. 16E, G): 0.61 long, 0.42 wide; yellowish. ***Eyes*** as in Fig. 16D, E. Clypeus as in Fig. 16D, G. Sternum as in Fig. 16F. ***Mouthparts*** (Figs 16F, 18C): labium rounded, not fused to sternum, anterior margin not indented at middle; endites strongly sclerotized on outer margin, with serrula in single row, a deep excavation and two large denticles. ***Abdomen*** ovoid, 0.59 long; dorsum soft portions pale white, without color pattern. Legs: yellow, without color pattern; femur IV thickened, wider than femora I−III. ***Palp*** (Fig. 18A, B, D, E): tibia not enlarged, length/width = 2.08, cymbium elongated ovoid; bulb pear-shaped in lateral view, with ventral side strongly protruding proximally, ca 2.34× as wide as tibia; the sperm duct with three loops in prolateral view; embolus tapered, with a sub-apical crest and a wrinkle.

**Figure 17.**
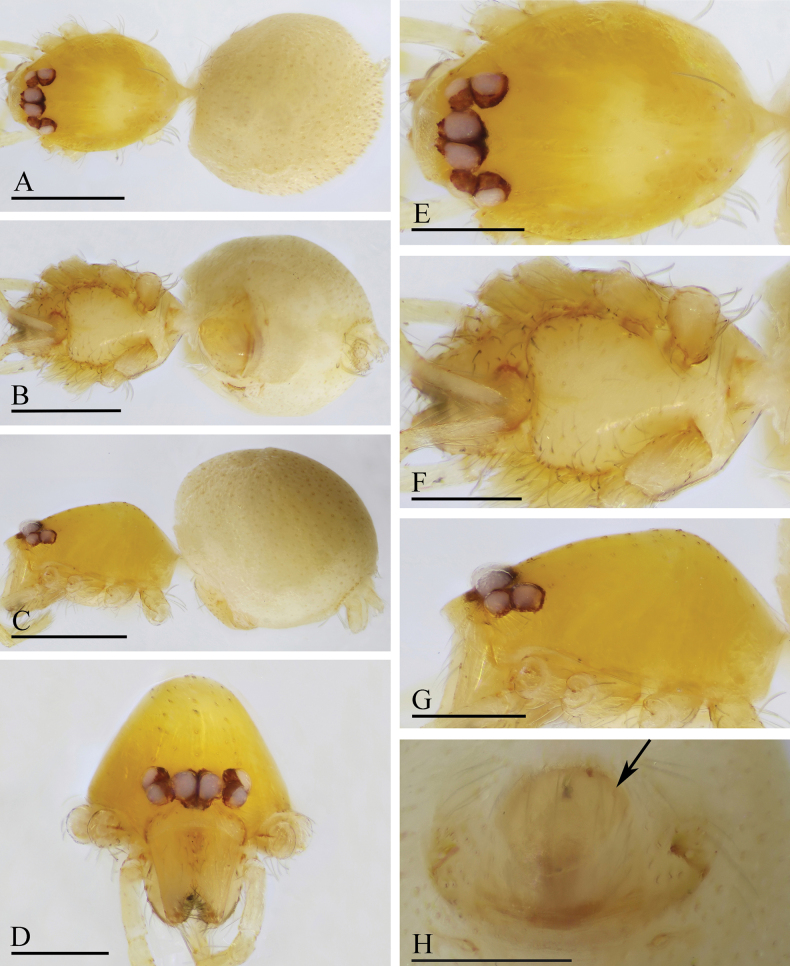
*Orchestina subconcava* sp. nov., paratype female. A–C. Habitus, dorsal, ventral and lateral views; D–G. Prosoma, anterior, dorsal, ventral and lateral views; H. Epigaster, ventral view, arrow shows the half tent-shaped marking. Scale bars: 0.4 mm (A–C); 0.2 mm (D–H).

**Female** (SYNU-1548). Same as male except as noted. ***Body***: habitus as in Fig. 17A–C; body length 1.29. ***Carapace*** (Fig. 17E, G): 0.61 long, 0.43 wide. ***Abdomen***: 0.69 long. ***Epigaster*** (Fig. 17H): without special external features, with a half tent-shaped marking visible through cuticle.

**Figure 18.**
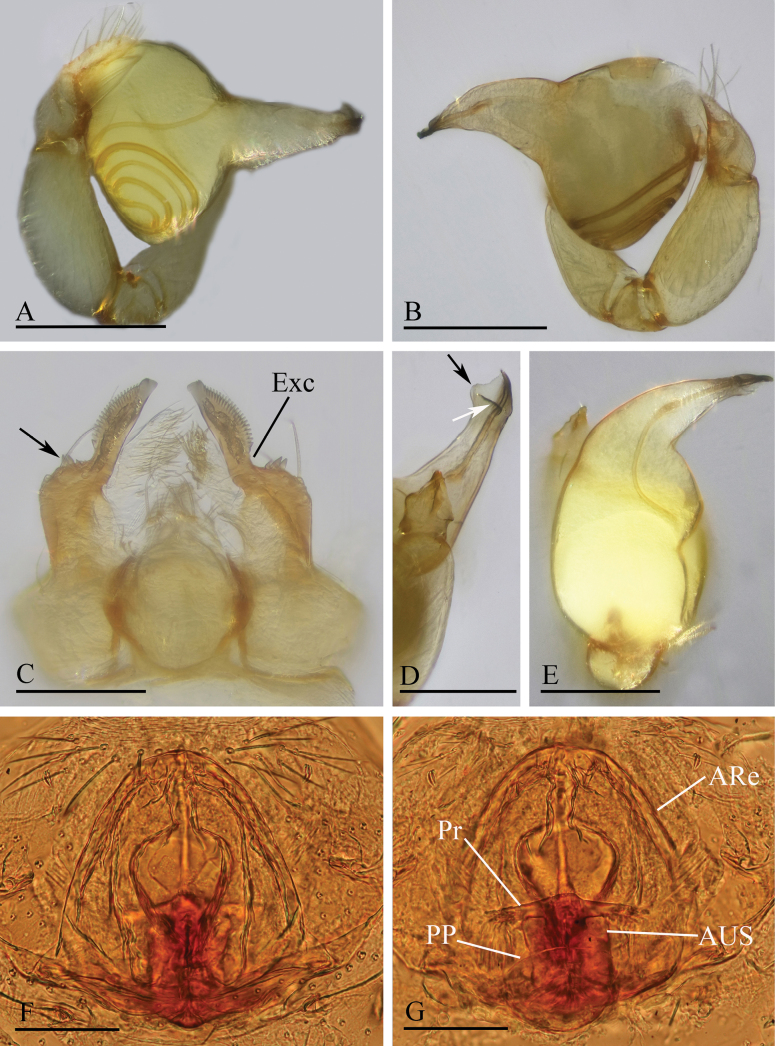
*Orchestina subconcava* sp. nov. A, B, E. Left palp, prolateral, retrolateral and dorsal views; C. Endites and labium, ventral view, arrow shows the large denticles; D. Distal part of bulb, prolateral view, black arrow shows the crest and white arrow shows the wrinkle; F, G. Endogyne, ventral and dorsal views. Abbreviations: ARe = anterior receptaculum; AUS = anterior uterine sclerite; Exc = excavations; PP = posterior plate; Pr = protrusion. Scale bars: 0.2 mm (A, B, E); 0.1 mm (C, D, F, G).

***Endogyne*** (Fig. 18F, G): with short medial clavate sclerite (AUS), provided with pair of short lateral protrusions (Pr); anterior receptaculum (ARe) rounded, semitransparent, distinctly longer than AUS; posterior plate (PP) present, large.

##### Distribution.

Known only from the type locality.

##### Etymology.

The specific name refers to the similarities to *Orchestina concava* Tong & Li, 2024.

#### ﻿ Orchestina sublongituba

Taxon classificationAnimalia

Tong & Li sp. nov.

9364F7F3-B5BB-5DEF-8D04-BB00D46EDA7D

https://zoobank.org/48553A00-827B-42E7-BA9C-642ED9D36666

Figs 19 , 20 , 21

##### Type material.

***Holotype*** China • ♂ (SYNU-1597), fogging; Yunnan, Mengla Co., Menglun Town, XNNR, 200 m east of the Lvshilin, limestone monsoon rainforest; 21°54.769'N, 101°16.959'E, 599 ± 14 m; 9.VIII.2011; Zheng G., Zhao Q. & Gao C. leg. ***Paratypes*.** China • 2 ♂ (SYNU-1598–99), same data as holotype • 2 ♂ (SYNU-F-4076–77), same data as holotype • 1 ♂ (SYNU-1600), fogging; XNNR, 48 Km.; 21°58.704'N, 101°19.748'E, 1088 ± 12 m; 12.VIII.2011; Zhao Q. & Chen Z. leg. • 3 ♀ (SYNU-1559–61), same data as above.

##### Additional material.

China • 4 ♂ (SYNU-F-4007–10), fogging; XNNR, 48 Km.; 21°58.704'N, 101°19.748'E, 1088 ± 12 m; 12.VIII.2011; Zhao Q. & Chen Z. leg. • 5 ♂ (SYNU-F-4012–16), same data as above • 3 ♂ 1 ♀ (SYNU-F-4027–30), same data as above • 1 ♂ (SYNU-F-4072), same data as above • 4 ♂ (SYNU-F-4085–88), same data as above • 2 ♀ (SYNU-1563–64), same data as above • 6 ♀ (SYNU-F-4328–33), same data as above • 4 ♀ (SYNU-F-4318–21), fogging; XNNR, 55 Km., secondary forest; 21°57.978'N, 101°12.167'E, 842 ± 14 m; 18.VIII.2011; Zheng G., Zhao Q. & Gao C. leg. • 1 ♂ (SYNU-F-4018), same data as above, • 2 ♂ (SYNU-F-4032–33), fogging; XNNR, 48 Km.; 21°58.764'N, 101°09.748'E, 1038 ± 12 m; 10.VIII.2011; Zhao Q. & Chen Z. leg. • 21 ♂ (SYNU-F-4040–60), same data as above • 1 ♂ (SYNU-F-4011), fogging; XNNR, 55 Km., the valley forest beside the artificial fishpond; 21°57.883'N, 101°12.147'E, 839 ± 18 m; 15.VIII.2011; Zheng G., Zhao Q. & Gao C. leg. • 6 ♂ (SYNU-F-4061–66), fogging; XNNR, 55 Km., about 1 km up the hill, seasonal rainforest; 21°57.949'N, 101°12.247'E, 788 ± 20 m; 13.VIII.2011; Zheng G., Zhao Q. & Gao C. leg. • 6 ♂ (SYNU-F-4034–39), fogging; XNNR, 55 Km., about 1 km up the hill, seasonal rainforest; 21°57.935'N, 101°12.305'E, 781 ± 17 m; 13.VIII.2011; Zheng G., Zhao Q. & Gao C. leg. • 3 ♂ (SYNU-F-4073–75), same data as above • 1 ♂ 3 ♀ (SYNU-F-4019–22), fogging; Xiaolongha Vill., Biodiversity corridor, seasonal rainforest; 21°24.213'N, 101°36.995'E, 834 ± 15 m; 3.VII.2012; Zhao Q. & Chen Z. leg. • 1 ♂ (SYNU-F-4031), fogging; Jinghong City, Guanping Town, seasonal rainforest; 21°13.353'N, 100°53.251'E, 832 ± 12 m; 21.VII.2012; Zhao Q. & Chen Z. leg. • 14 ♂ (SYNU-F-4067–70), fogging; XNNR, 200 m east of the Lvshilin, limestone monsoon rainforest; 21°54.617'N, 101°16.843'E, 738 ± 17 m; 7.VIII.2011; Zheng G., Zhao Q. & Gao C. leg. • 1 ♂ (SYNU-F-4089), fogging; XTBG, Ficus microcarpa forest; 21°34.284'N, 101°15.986'E, 538 ± 9 m; 22.VIII.2011; Zheng G., Zhao Q. & Gao C. leg.

##### Diagnosis.

The new species is similar to *O. codalmasi* Wunderlich, 2011 (female unknown) and *O. longituba* sp. nov. in the strongly coiled sperm duct and the enlarged palpal tibia, but can be distinguished from *O. codalmasi* by the very long tube-shaped embolus (vs short; cf. Fig. 21A, B, D and Wunderlich 2008: fig. 7); from *O. longituba* by palpal tibia wider than bulbus (vs narrower; cf. Fig. 21A, B and Fig. 12A, B), the tongue-shaped labium (vs diamond-shaped; cf. Fig. 21C and Fig. 12C) and the saddle-shaped marking on epigaster (vs lacking; cf. Fig. 20H and Fig. 11H).

##### Description.

**Figure 19.**
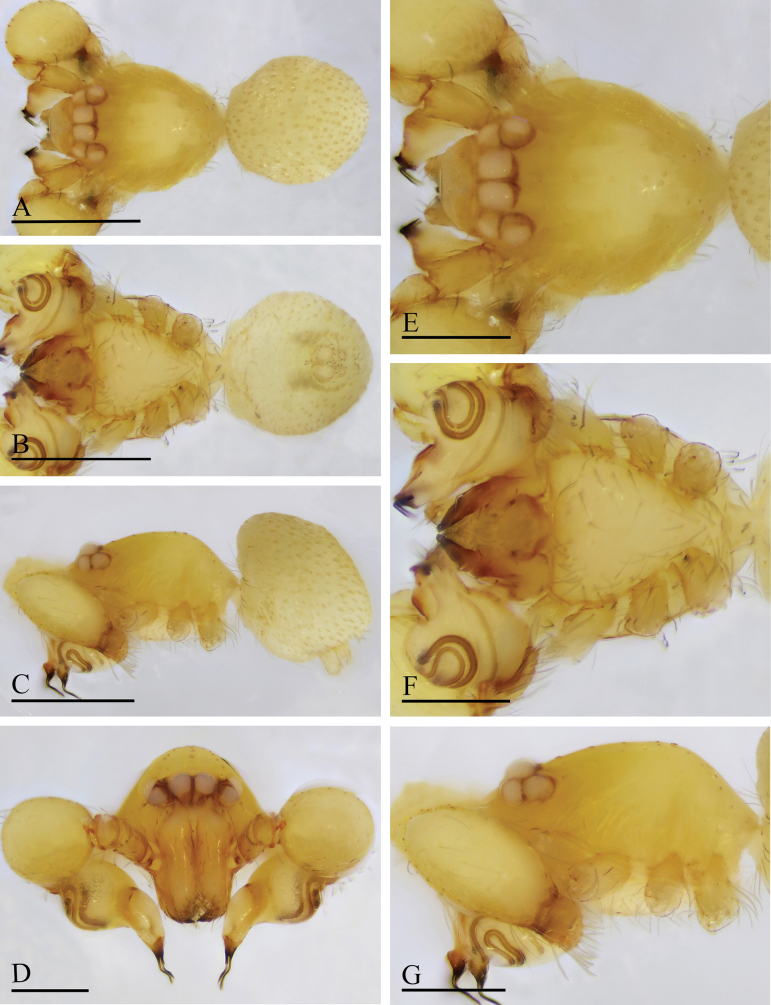
*Orchestina sublongituba* sp. nov., holotype male. A–C. Habitus, dorsal, ventral and lateral views; D–G. Prosoma, anterior, dorsal, ventral and lateral views. Scale bars: 0.4 mm (A–C); 0.2 mm (D–G).

**Male** (holotype). ***Body***: habitus as in Fig. 19A–C; body length 1.05. ***Carapace*** (Fig. 19E, G): 0.58 long, 0.41 wide. ***Eyes*** as in Fig. 19D, E. Clypeus as in Fig. 19D. ***Sternum*** (Fig. 19F): longer than wide. ***Mouthparts*** (Figs 19F, 21C): labium tongue-shaped, not fused to sternum; endites strongly sclerotized, with serrula in single row, outer margin smoothly curved. ***Abdomen***: ovoid, 0.58 long; dorsum soft portions pale white, without color pattern. ***Legs***: yellow, without color pattern; femur IV thickened, wider than femora I−III. ***Palp*** (Fig. 21A, B, D, E): tibia strongly enlarged, length/width = 1.49, cymbium elongated ovoid; bulb pear-shaped in lateral view, with ventral side strongly protruding proximally, ca 0.99× as wide as tibia; the sperm duct with two loops in prolateral view; embolus long tube-shaped, with a basal node (Fig. 21A, B).

**Figure 20.**
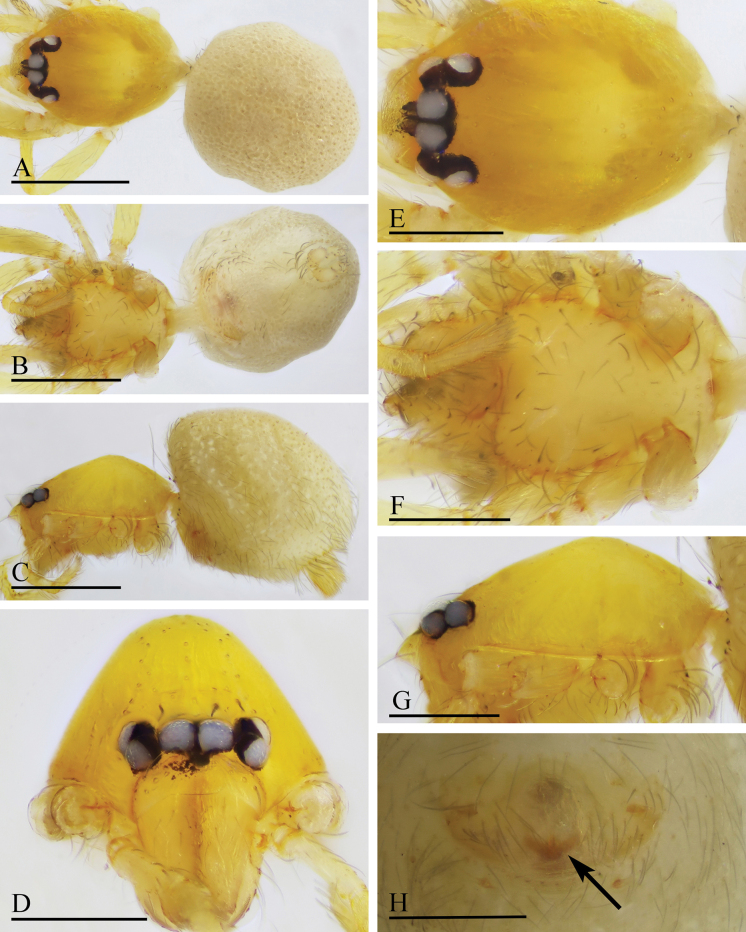
*Orchestina sublongituba* sp. nov., paratype female. A–C. Habitus, dorsal, ventral and lateral views; D–G. Prosoma, anterior, dorsal, ventral and lateral views; H. Epigaster, ventral view, arrow shows the marking. Scale bars: 0.4 mm (A–C); 0.2 mm (D–H).

**Female** (SYNU-1559). Same as male except as noted. ***Body***: habitus as in Fig. 20A–C; body length 1.20. ***Carapace*** (Fig. 20E, G): 0.59 long, 0.42 wide. ***Abdomen***: 0.62 long. ***Epigaster*** (Fig. 20H): without special external features, with a saddle-shaped marking visible through cuticle.

**Figure 21.**
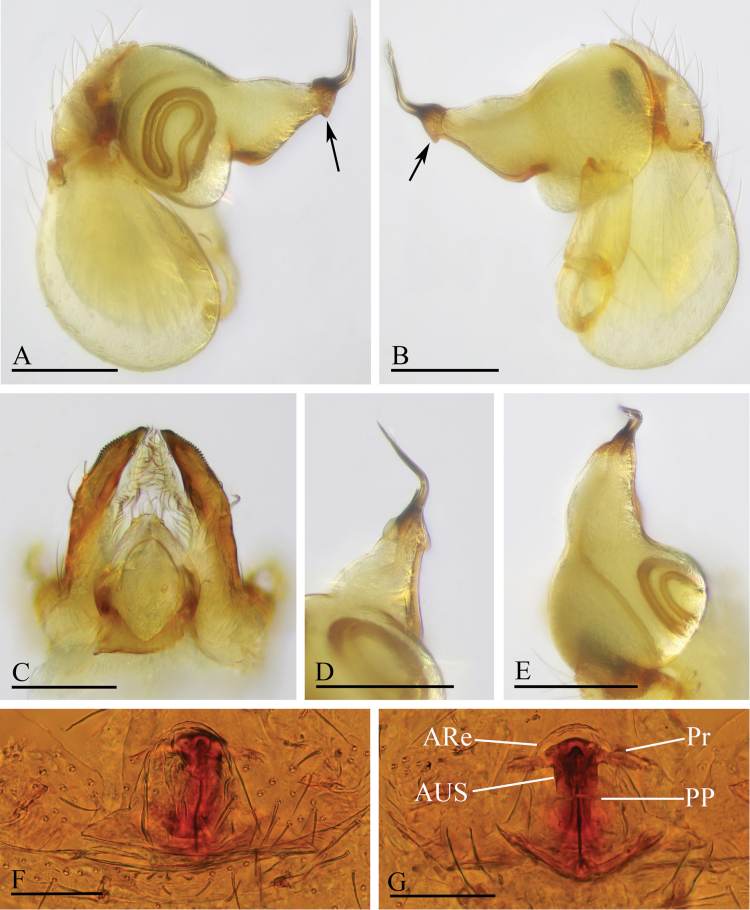
*Orchestina sublongituba* sp. nov. A, B, E. Left palp, prolateral, retrolateral and dorsal views, arrows in Fig. A, B show the node; C. Endites and labium, ventral view; D. Distal part of bulb, prolateral view; F, G. Endogyne, ventral and dorsal views. Abbreviations: ARe = anterior receptaculum; AUS = anterior uterine sclerite; PP = posterior plate; Pr = protrusion. Scale bars: 0.2 mm (A, B, D, E); 0.1 mm (C, F, G).

***Endogyne*** (Fig. 21F, G): with stout medial clavate sclerite (AUS), provided with pair of long lateral protrusions (Pr); anterior receptaculum (ARe) rounded, semitransparent, slightly longer than AUS; posterior plate (PP) present, large.

##### Distribution.

Known only from the type locality.

##### Etymology.

The specific name refers to the similarities to *O. longituba* sp. nov.

#### ﻿ Orchestina tentoria

Taxon classificationAnimalia

Tong & Li sp. nov.

8EBB26FB-BFA5-5E3E-9034-0EA028FE0EA0

https://zoobank.org/43B51F79-9F61-42A5-99BA-C5E1DF710471

Figs 22 , 23 , 24

##### Type material.

***Holotype*** China • ♂ (SYNU-1579), fogging; Yunnan, Mengla Co., Menglun Town, XNNR, 48 Km.; 21°58.704'N, 101°19.748'E, 1088 ± 12 m; 12.VIII.2011; Zhao Q. & Chen Z. leg. ***Paratypes*.** China • 2 ♂ (SYNU-1581–82), same data as holotype • 3 ♀ (SYNU-1553–55), same data as holotype • 1 ♀ (SYNU-1556), same data as holotype • 2 ♀ (SYNU-1557–58), same data as holotype • 1 ♂ (SYNU-1580), fogging; same data as holotype.

##### Additional material.

China • 3 ♂ (SYNU-F-3740–42), same data as holotype • 2 ♂ (SYNU-F-3747–48), same data as holotype • 4 ♂ (SYNU-F-3750–53), same data as holotype • 4 ♂ (SYNU-F-3766–69), same data as holotype • 2 ♂ (SYNU-F-3771–72), same data as holotype • 1 ♂ (SYNU-F-3773), same data as holotype • 2 ♀ (SYNU-F-4269–70), same data as holotype • 6 ♀ (SYNU-F-4271–76), same data as holotype • 7 ♀ (SYNU-F-4280–86), same data as holotype • 1 ♀ (SYNU-F-4302), same data as holotype • 1 ♀ (SYNU-F-4303), same data as holotype • 12 ♂ (SYNU-F-3754–65), fogging; XNNR, 48 Km.; 21°58.764'N, 101°09.748'E, 1038 ± 12 m; 10.VIII.2011; Zhao Q. & Chen Z. leg. • 1 ♂ (SYNU-F-4004), same data as above • 2 ♀ (SYNU-F-4267–68), same data as above • 15 ♀ (SYNU-F-4287–4301), same data as above • 4 ♂ (SYNU-F-3743–46), fogging; XNNR, 55 Km., about 1 km up the hill, seasonal rainforest; 21°57.935'N, 101°12.305'E, 781 ± 17 m; 13.VIII.2011; Zheng G., Zhao Q. & Gao C. leg. • 2 ♀ (SYNU-F-4277–78), same data as above • 1 ♀ (SYNU-F-4279), fogging; XNNR, 55 Km., about 1 km up the hill, seasonal rainforest; 21°57.949'N, 101°12.247'E, 788 ± 20 m; 13.VIII.2011; Zheng G., Zhao Q. & Gao C. leg. • 1 ♀ (SYNU-F-4266), fogging; Nanshahe Vill., seasonal rainforest; 21°36.201'N, 101°34.398'E, 826 ± 43 m; 14.VII.2012; Zhao Q. & Chen Z. leg.

##### Diagnosis.

The new species is similar to *Orchestina bialata* Liu, Xiao & Xu, 2016 in the enlarged palpal tibiae and the shape of male endites, but can be distinguished by the sperm duct with five loops (vs four loops; cf. Fig. 24A and Liu et al. 2019: fig. 7A), the tongue-shaped labium (vs diamond-shaped; cf. Fig. 23C and Liu et al. 2019: figs 6F, 8D) and the tent-shaped marking of female epigaster (vs triangular; cf. Fig. 23H and Liu et al. 2016: fig. 5F).

##### Description.

**Figure 22.**
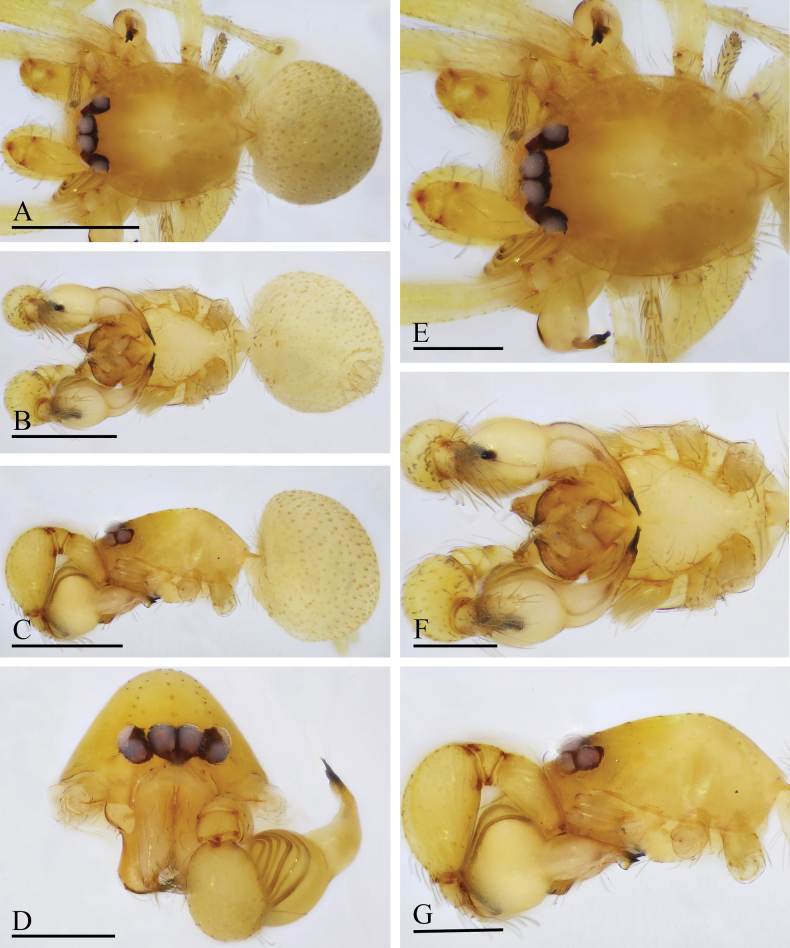
*Orchestina tentoria* sp. nov., holotype male. A–C. Habitus, dorsal, ventral and lateral views; D–G. Prosoma, anterior, dorsal, ventral and lateral views. Scale bars: 0.4 mm (A–C); 0.2 mm (D–G).

**Male** (holotype). ***Body***: habitus as in Fig. 22A–C; body length 1.28. ***Carapace*** (Fig. 22E, G): 0.57 long, 0.45 wide. ***Eyes*** as in Fig. 22D, E. ***Clypeus*** as in Fig. 22D. ***Sternum*** as in Fig. 22F. ***Mouthparts*** (Figs 22D, F, 24C): labium tongue-shaped, not fused to sternum, anterior margin not indented at middle; endites strongly sclerotized, with serrula in single row, outer margin strongly curved. ***Abdomen*** ovoid, 0.59 long; dorsum soft portions pale white, without color pattern. ***Legs***: yellow, without color pattern; femur IV thickened, wider than femora I−III. ***Palp*** (Fig. 24A, B, D, E): tibia strongly enlarged, length/width = 2.01, cymbium elongated ovoid; bulb pear-shaped in lateral view, with ventral side strongly protruding proximally, ca 1.36× as wide as tibia; the sperm duct with five loops in prolateral view; embolus short, with sub-apical, opaque crest (Fig. 24A, B).

**Figure 23.**
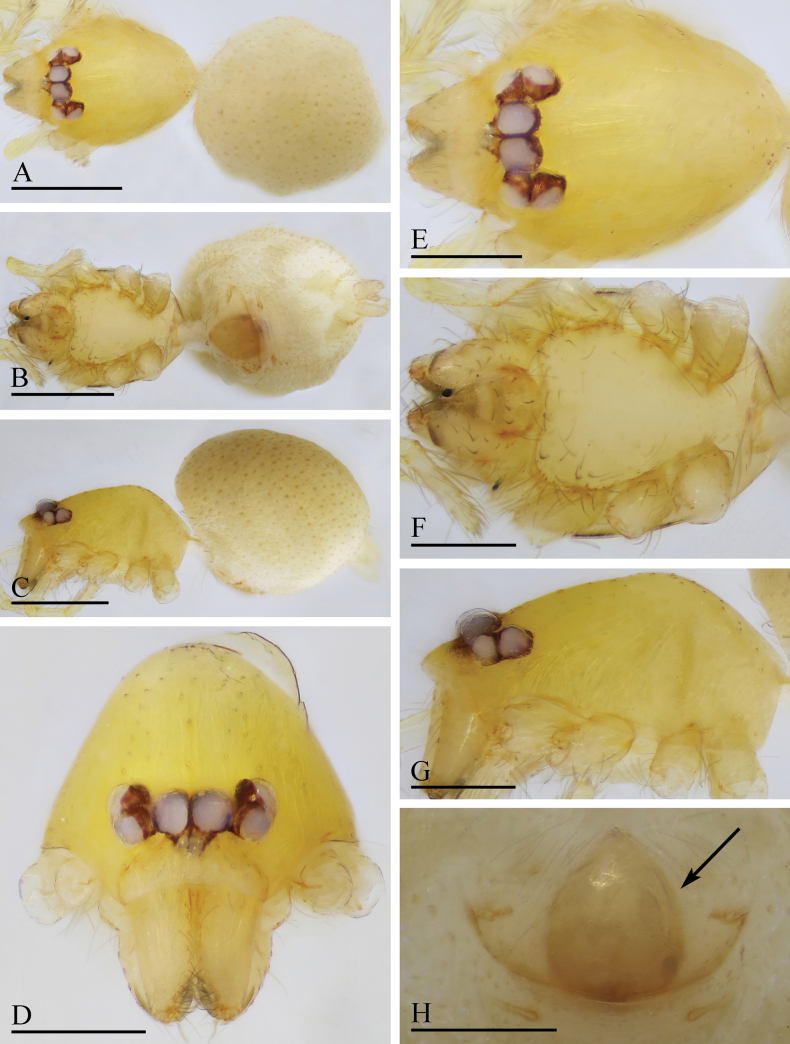
*Orchestina tentoria* sp. nov., paratype female. A–C. Habitus, dorsal, ventral and lateral views; D–G. Prosoma, anterior, dorsal, ventral and lateral views; H. Epigaster, ventral view, arrow shows the tent-shaped marking. Scale bars: 0.4 mm (A–C); 0.2 mm (D–H).

**Female** (SYNU-1553). Same as male except as noted. ***Body***: habitus as in Fig. 23A–C; body length 1.31. ***Carapace*** (Fig. 23E, G): 0.64 long, 0.47 wide. ***Abdomen***: 0.68 long. ***Epigaster*** (Fig. 23H): without special external features, with a tent-shaped marking visible through cuticle.

**Figure 24.**
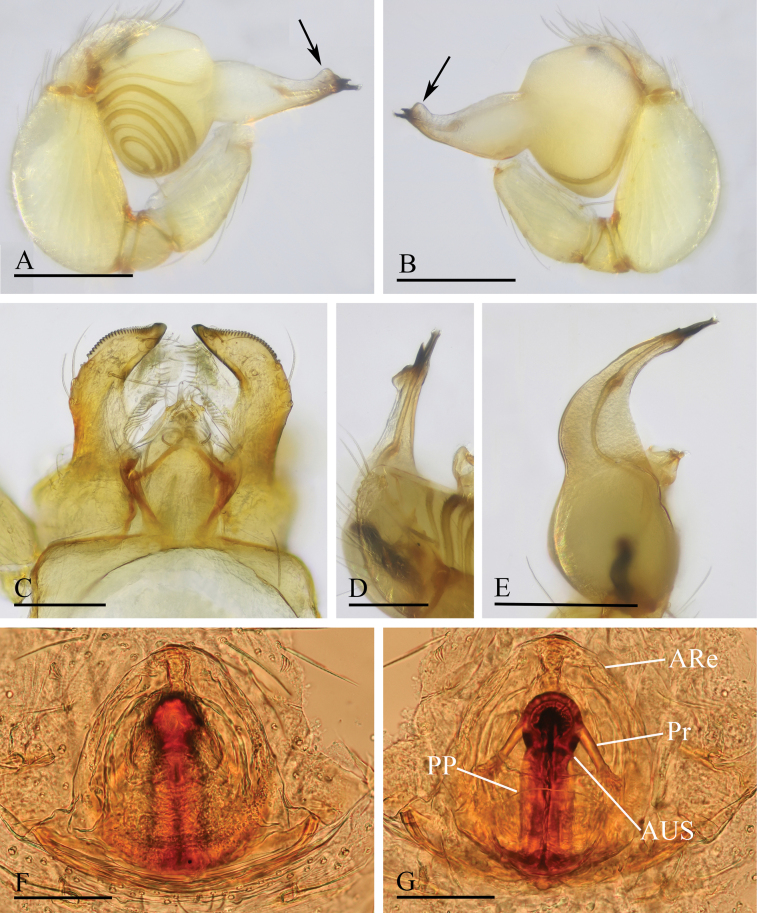
*Orchestina tentoria* sp. nov. A, B, E. Left palp, prolateral, retrolateral and dorsal views, arrows in Fig. A, B show the crest; C. Endites and labium, ventral view; D. Distal part of bulb, prolateral view; F, G. Endogyne, ventral and dorsal views. Abbreviations: ARe = anterior receptaculum; AUS = anterior uterine sclerite; PP = posterior plate; Pr = protrusion. Scale bars: 0.2 mm (A, B, E); 0.1 mm (C, D, F, G).

***Endogyne*** (Fig. 24F, G): with long medial clavate sclerite (AUS), provided with pair of long lateral protrusions (Pr); lateral protrusions at angle of about 30° with medial clavate sclerite; anterior receptaculum (ARe) rounded, semitransparent, slightly longer than AUS; posterior plate (PP) present, large.

##### Distribution.

Known only from the type locality.

##### Etymology.

The specific name is derived from the Latin tent and refers to the tent-shaped marking on epigaster of female.

#### ﻿ Orchestina xuexing

Taxon classificationAnimalia

Tong & Li sp. nov.

B6D385DE-AEAA-510D-ADDA-5439F420E36D

https://zoobank.org/9426D7A0-DB18-4B71-B10F-6212DBFD4CEE

Figs 25 , 26 , 27

##### Type material.

***Holotype*** China • ♂ (SYNU-1594), fogging; Yunnan, Mengla Co., Menglun Town, XNNR, 48 Km., artificial forest; 21°53.997'N, 101°16.957'E, 593 ± 18 m; 11.VIII.2011; Zheng G., Zhao Q. & Gao C. leg. ***Paratypes*.** China • 2 ♂ (SYNU-1595–96), same data as holotype • 1 ♀ (SYNU-F-4730), fogging; Huigang Vill., Xilu habitat restoration area, monsoon forest; 21°37.045'N, 101°35.268'E, 764 ± 25 m; 12.VII.2012; Zhao Q. & Chen Z. leg. • 1 ♀ (SYNU-F-4361), same data as above.

##### Additional material.

China • 1 ♀ (SYNU-F-4369), fogging; XNNR, 55 Km., the secondary forest on the mountain top; 21°57.978'N, 101°12.167'E, 842 ± 14 m; 18.VIII.2011; Zheng G., Zhao Q. & Gao C. leg. • 2 ♀ (SYNU-F-4666–67), fogging; XNNR, 48 Km.; 21°58.764'N, 101°09.748'E, 1038 ± 12 m; 10.VIII.2011; Zhao Q. & Chen Z. leg.

##### Diagnosis.

The new species is similar to *Orchestina manicata* Simon, 1893 in the enlarged palpal tibiae and the pear-shaped bulb, but can be distinguished by the shape of embolus, which is boot-shaped in lateral view and straight in dorsal view (vs triangular bifid; cf. Fig. 27A, B, D and Ranasinghe and Benjamin 2025: figs 3D, 4A), the smoothly curved outer margin of male endites (vs straight; cf. Fig. 27C and Ranasinghe and Benjamin 2025: fig. 3D) and the long medial clavate sclerite of endogyne (vs short; cf. Fig. 27F and Ranasinghe and Benjamin 2025: fig. 4B).

##### Description.

**Figure 25.**
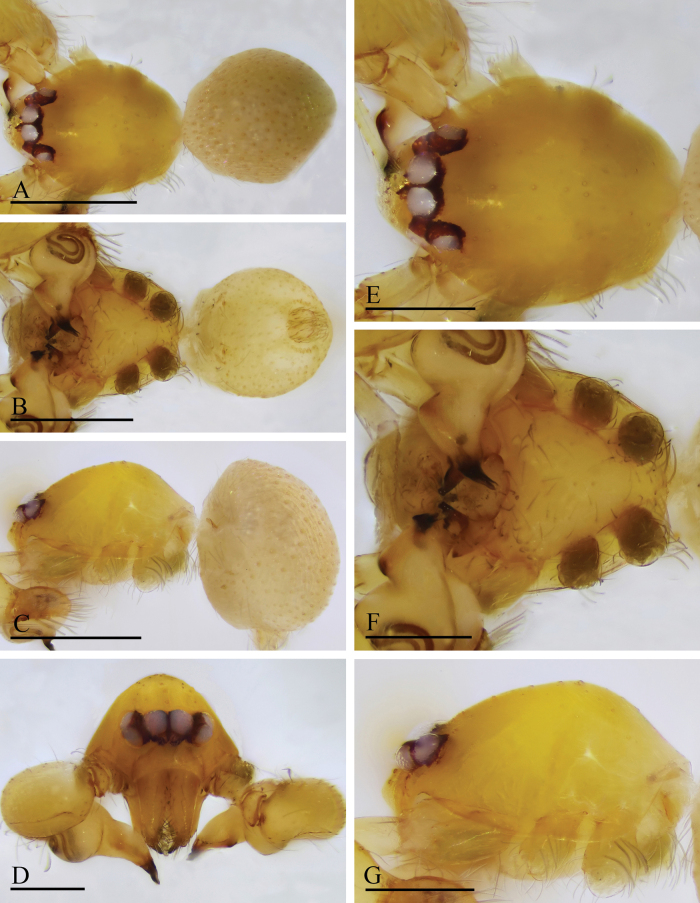
*Orchestina xuexing* sp. nov., holotype male. A–C. Habitus, dorsal, ventral and lateral views; D–G. Prosoma, anterior, dorsal, ventral and lateral views. Scale bars: 0.4 mm (A–C); 0.2 mm (D–G).

**Male** (holotype). ***Body***: habitus as in Fig. 25A–C; body length 1.17. ***Carapace*** (Fig. 25E, G): 0.57 long, 0.43 wide; yellowish, oval in dorsal view, without net-shaped pattern. ***Eyes*** as in Fig. 25D, E. ***Clypeus*** as in Fig. 25D, G. ***Sternum*** as in Fig. 25F. ***Mouthparts*** (Figs 25D, F, 27C): labium diamond-shaped, not fused to sternum; endites with serrula in single row, outer margin smoothly curved. ***Abdomen*** ovoid, 0.51 long; dorsum soft portions pale white, without color pattern. Legs: yellow, without color pattern; femur IV thickened, wider than femora I−III. ***Palp*** (Fig. 27A, B, D): tibia strongly enlarged, length/width = 1.46, cymbium elongated ovoid; bulb pear-shaped in lateral view, with ventral side strongly protruding proximally, ca 1.07× as wide as tibia; the sperm duct with two loops in prolateral view; embolus short, boot-shaped.

**Figure 26.**
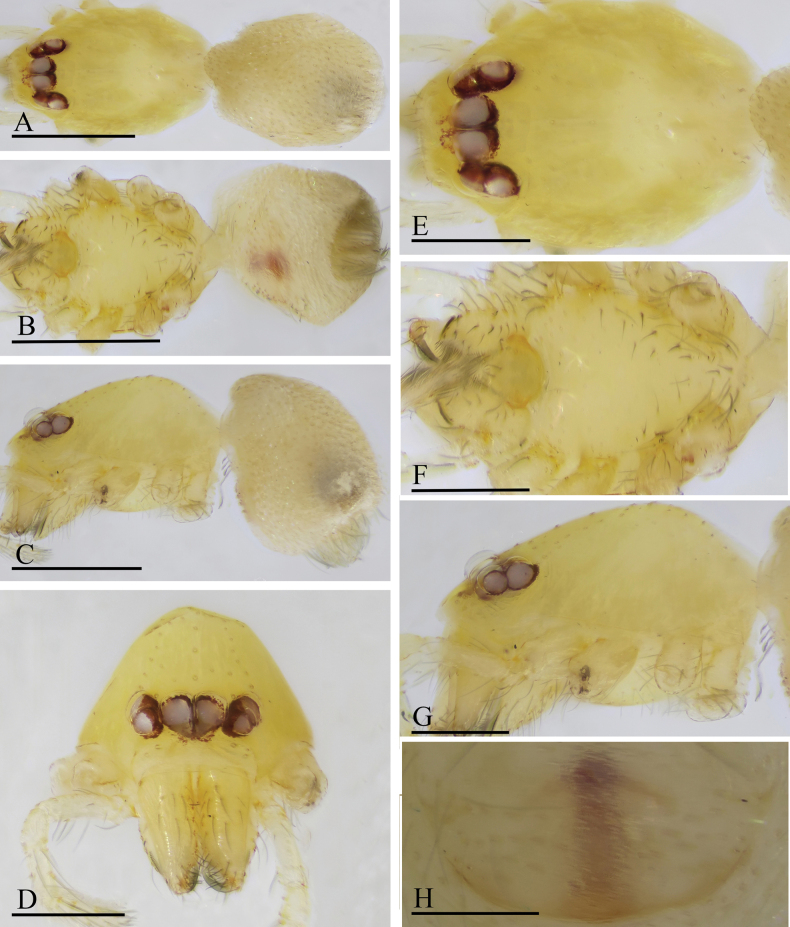
*Orchestina xuexing* sp. nov., paratype female. A–C. Habitus, dorsal, ventral and lateral views; D–G. Prosoma, anterior, dorsal, ventral and lateral views; H. Epigaster, ventral view. Scale bars: 0.4 mm (A–C); 0.2 mm (D–G); 0.1mm (H).

**Female** (SYNU-F-4730). Same as male except as noted. ***Body***: habitus as in Fig. 26A–C; body length 1.21. ***Carapace*** (Fig. 26E, G): 0.67 long, 0.42 wide. ***Abdomen***: 0.55 long. ***Epigaster*** (Fig. 26H): without special external features.

**Figure 27.**
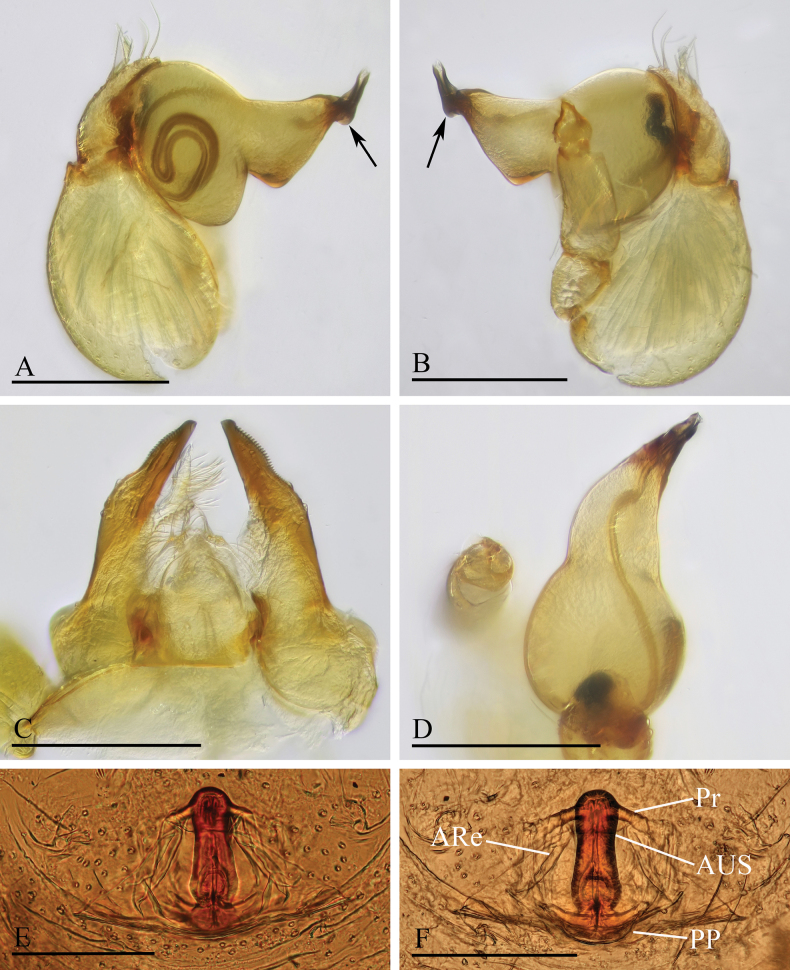
*Orchestina xuexing* sp. nov. A, B, D. Left palp, prolateral, retrolateral and dorsal views, arrows show the boot-shaped embolus; C. Endites and labium, ventral view; E, F. Endogyne, ventral and dorsal views. Abbreviations: ARe = anterior receptaculum; AUS = anterior uterine sclerite; PP = posterior plate; Pr = protrusion. Scale bars: 0.2 mm (A, B, D); 0.1 mm (C, E, F).

***Endogyne*** (Fig. 27E, F): with long medial clavate sclerite (AUS), provided with pair of short lateral protrusions (Pr); lateral protrusions at angle of about 75° with medial clavate sclerite; anterior receptaculum (ARe) triangular, semitransparent, slightly longer than AUS; posterior plate (PP) present, large.

##### Distribution.

Known only from the type locality.

##### Etymology.

The specific name is from Chinese Pinyin, meaning boot-shaped, and refers to the boot-shaped embolus.

## Supplementary Material

XML Treatment for Orchestina alata

XML Treatment for Orchestina aureola

XML Treatment for Orchestina caixiaae

XML Treatment for Orchestina longituba

XML Treatment for Orchestina qingyuani

XML Treatment for Orchestina subconcava

XML Treatment for Orchestina sublongituba

XML Treatment for Orchestina tentoria

XML Treatment for Orchestina xuexing

## ﻿Additional information

### Use of AI

No use of AI was reported.

### Author ORCIDs

Chenxue Song https://orcid.org/0009-0005-2291-1870

Dongju Bian https://orcid.org/0000-0002-9324-8026

Yanfeng Tong https://orcid.org/0000-0002-4348-7029

Shuqiang Li https://orcid.org/0000-0002-3290-5416

### Data availability

All of the data that support the findings of this study are available in the main text.
